# Rigid DNA Beams for High-Resolution Single-Molecule Mechanics**

**DOI:** 10.1002/anie.201302727

**Published:** 2013-06-21

**Authors:** Emanuel Pfitzner, Christian Wachauf, Fabian Kilchherr, Benjamin Pelz, William M Shih, Matthias Rief, Hendrik Dietz

**Affiliations:** Physik Department, Walter Schottky Institute, Technische Universität MünchenAm Coulombwall 4a, 85748 Garching near Munich (Germany) E-mail: dietz@tum.de Homepage: http://bionano.physik.tu-muenchen.de; Physik Department, Lehrstuhl für Biophysik, Technische Universität MünchenJames-Franck-Strasse 1, Garching near Munich (Germany); Dana-Farber Cancer Institute, Harvard Medical School44 Binney Street, Boston, MA 02115 (USA)

**Keywords:** biophysics, DNA nanotechnology, DNA structures, force spectroscopy, single-molecule experiments

Single molecule mechanical techniques like AFM or optical tweezers provide insight into the conformational dynamics of macromolecules and allow reconstructing details of the free energy landscapes that direct such processes.^1^ Single-molecule mechanical assays have been successfully applied to analyze large conformational changes like the ones that occur in protein unfolding or in the motion of molecular motors. However, conformational transitions in many native proteins involve much smaller length changes, on the order of a nanometer or less.^2^ Conventional force spectroscopy at such fine resolution is affected by significant signal-to-noise limitations in the regime of low forces (less than 10 pN). Yet it is precisely the regime of low forces that deserves attention, because the functionally relevant conformational dynamics of proteins and other biological macromolecules are located here.

In a typical single-molecule mechanical assay, the molecule of interest is attached to a sensitive probe such as two micron-sized beads held in optical tweezers (Figure 1 A). To avoid non-specific interactions with the surface of the probe, the attachment of the molecule of interest to the probe typically occurs through molecular linkers such as double-stranded DNA (dsDNA).^3^ The mechanical stiffness of those linkers is critical for the signal-to-noise ratio of the measurement.^4^ This can be modeled through Monte Carlo simulations using the consideration that every degree of freedom in a thermodynamic ensemble has the energy *k*_B_ *T*/2. For soft flexible linkers, thermal forces will drive the probe through large displacements, but stiff linkers clamp the probe and suppress unwanted noise (Figure 1 B,C).

**Figure 1:**
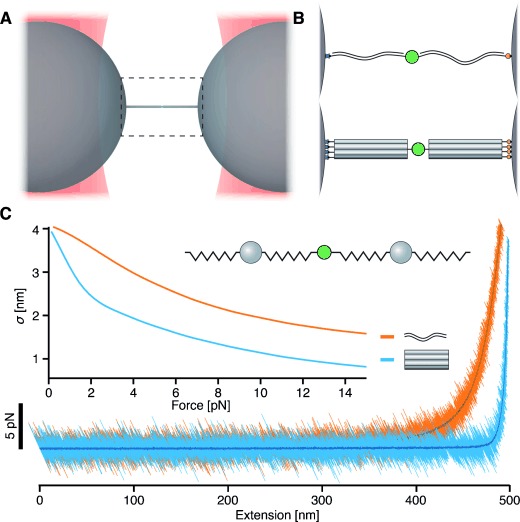
Noise suppression for optical-tweezer experiments. A) Scheme of two 1 µm beads held in laser traps tethered by a 500 nm linker (drawn to scale). B) Magnification of the “reaction chamber”. Top: a conventional linker system comprising two dsDNA molecules attached to the molecule of interest (green circle). Bottom: a stiff linker system comprising multiple dsDNA molecules aligned in parallel. C) Monte-Carlo simulations were used to estimate the load-dependent fluctuation dynamics of two beads held in laser traps. One example of simulated force-extension trace for each linker system is shown and the averaged response from 200 simulated traces overlaid in a darker shade. Inset: simulated force-dependent noise amplitude as measured by the standard deviation of the extension for floppy versus stiff linkers.

Previously, noise suppression was achieved by taking advantage of the tensile stiffening of polymeric linkers upon stretching to high forces above 10 pN (Figure 1 C).^5^ Short (less than 50 nm) B-form DNA linkers were also tested for their ability to stiffen the connection between the beads,^6^ but the noise suppression was minor, presumably because of the unusual flexibility of short duplex DNA molecules^7^ and the flexibility of the DNA-bead attachment.^8^ Molecular self-assembly with DNA offers unique possibilities to create functional structures with user-defined shape and mechanical properties.^9^ Herein, we took advantage of this technique to establish rigid beam-like molecular linkers that enable the study of conformational transitions of single molecules with unprecedented resolution.

We tested four linker designs for rigid-beam-like mechanical behavior. We constructed helix bundles consisting of six, eight, ten, and twelve DNA double helices that were aligned and cross-linked in parallel at contour-length extension (Supporting Information, Figure S1).9c Because the bundles form from a DNA molecule 7560 bases in length, the bundle length decreases with increasing number of DNA double helices in the bundle. The bundles were self-assembled in one-pot thermal renaturation reactions, as previously described.9c Assembly was confirmed by agarose gel electrophoresis (Figure S2) and by direct imaging using negative-stain transmission electron microscopy (TEM; Figure S3). Single particle TEM micrographs were aligned against a randomly chosen reference particle micrograph using cross-correlation maximization within a 25 pixel radial interval centered in the middle of each bundle micrograph. Average bundle images were computed (Figure S4 A) that reflected decreasing shape fluctuations with increasing cross-sectional area of the bundles. The ten- and twelve-helix bundles in particular appeared as rigid beams with little shape variation. Particle backbone tracing in single-bundle micrographs was used to determine the contour lengths and end-to-end distances (Figure S4 B,C). This data agreed with predictions from a semi-flexible beam theory^10^ when assuming persistence lengths of 2 μm for the six-helix bundle and 3.5 μm for the eight-helix bundle. The value found for the six-helix bundle is consistent with previous results.^11^ For the ten- and twelve-helix bundles, the measured end-to-end distances were identical to the measured contour lengths within the resolution of the backbone tracing method. For all bundles, the measured average contour lengths matched the expected lengths to 2 % accuracy. The standard deviation from the average contour length was 3 % or less for all bundle types. We attributed fluctuations in the measured contour lengths mostly to limitations of backbone tracing, rather than actual absolute contour length fluctuations.

To analyze the mechanics of individual helix bundles in a dual-beam optical tweezer setup (Figure 2 A), the two opposing helical interfaces of the bundles were functionalized with multiple biotin- and digoxigenin-modified DNA oligonucleotides (see Figure S1), respectively, and then attached to streptavidin- and anti-digoxigenin-modified one micrometer silica beads. The force-extension responses of individual helix bundles (Figure 2 B) were as expected (Figure 1 C), except for a false impression of extensibility when stretched to the contour length (Figure 2 B). This data gave stretching stiffness values *k*_app_ that were an order of magnitude smaller than expected (*k*_expected_=*N* *K*/*L* where *L* is the contour length, *K* the stretch modulus of a single dsDNA,^12^ and *N* the number of helices in the bundle). However, the observed apparent bundle extensibility also correlated directly with the stiffness of the laser traps used in the experiments (Figure 2 C), thus pointing to errors in determining the absolute bead displacements rather than an actual significant stretching of the bundles beyond their unloaded contour length. These errors directly propagate into the quantity extension for the special case of linkers that are significantly stiffer than the laser traps themselves. Importantly, the force-extension data obtained, for example, with the ten-helix bundles had the desired noise suppression in the force regime from 1 to 10 pN (Figure 2 D). The noise suppression remained comparable when using two copies of the ten-helix bundles that flanked a short dsDNA element as a mimic for a molecule under study that lacks conformational dynamics (Figure 2 D). Because the ten-helix bundle combined attractive geometrical properties with an absolute length of approximately 250 nm and rigid-beam-like mechanical properties, we used it for the experiments that are described next. However, the other bundle types also offer noise suppression, with a slight trend toward greater noise suppression for thicker bundles (Figure S5).

**Figure 2:**
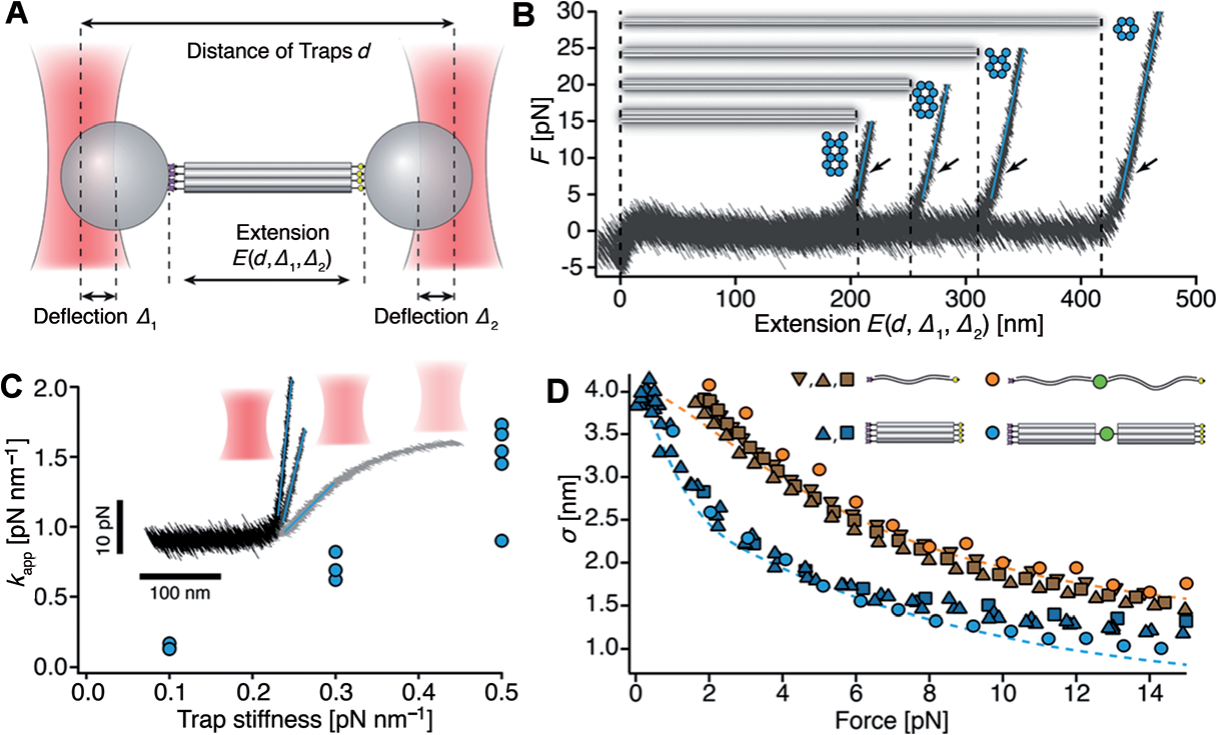
Establishing a stiff linker system. A) Experimental setup. B) Force-extension data obtained with six, eight, ten, and twelve-helix bundles. Apparent linear extensibility (*k*_app_) upon stretching the bundles beyond their contour lengths (arrows). C) Graph of *k*_app_ when varying the laser trap stiffness. Inset: force-extension response of an eight-helix-bundle linker at different values of trap stiffness. Darker shade indicates greater trap stiffness. D) Force-dependent noise amplitudes when using ten-helix bundles (cyan/blue) versus conventional dsDNA linkers (orange/brown). Dashed lines: theoretically predicted noise suppression (see Figure 1 C). Experiments were also carried out using double linkers that flank a short dsDNA bridging element (green circles).

For proof-of-concept purposes we compared the unfolding and refolding dynamics of a previously studied stable 20 base-pair (bp) long DNA hairpin^13^ using conventional dsDNA linkers (Figure 3 A,B) versus using stiff ten-helix bundle linkers (Figure 3 C,D; see Figure S6 for design details). The data were consistent with each other (see also Figure S7), except for a larger separation between the two dominant deflection states corresponding to the unfolded and folded states of the hairpin in the stiff linker data (Figure 3 B versus Figure 3 D), which is an expected consequence of the inextensibility of the ten-helix bundle linkers. We also determined the force-dependent unfolding- and refolding-transition rate constants of the 20 bp hairpin (Figure 3 E) by analyzing the distribution of dwell times in the constant-distance data (see also Figure S7). The rate constants obtained from the experiments with the stiff linker system agreed well—within experimental error—with those from our reference experiments and also with previous experiments13a that were both performed with the conventional dsDNA linker system. Notably, the noise suppression that was supplied by the stiff linker system even at the relatively high force load of around 14 pN gave access to more detailed information about the energy landscape that directs the hairpin transition. Histograms of the deflection states that were tested by the combined system of beads, linkers, and hairpin when the traps are set to a constant distance revealed transiently populated substates for the stiff linkers, while these substates were masked by noise in the experiments with the conventional dsDNA linkers (Figure 3 F). These distributions allow for reconstruction of the energy landscape that governs the hairpin transitions, in which the landscape derived from the data obtained with the stiff linkers now offers more details (such as sharper barriers) owing to the enhanced resolution (Figure 3 G). Reconvolution of the higher-resolution energy landscape with the broader noise characteristics of the conventional dsDNA linkers gave deflection distributions that were consistent with the distributions that we measured using the conventional linkers (Figure S8), which suggests that the higher resolution features indeed could not have been extracted when using the noisier dsDNA linkers.

**Figure 3:**
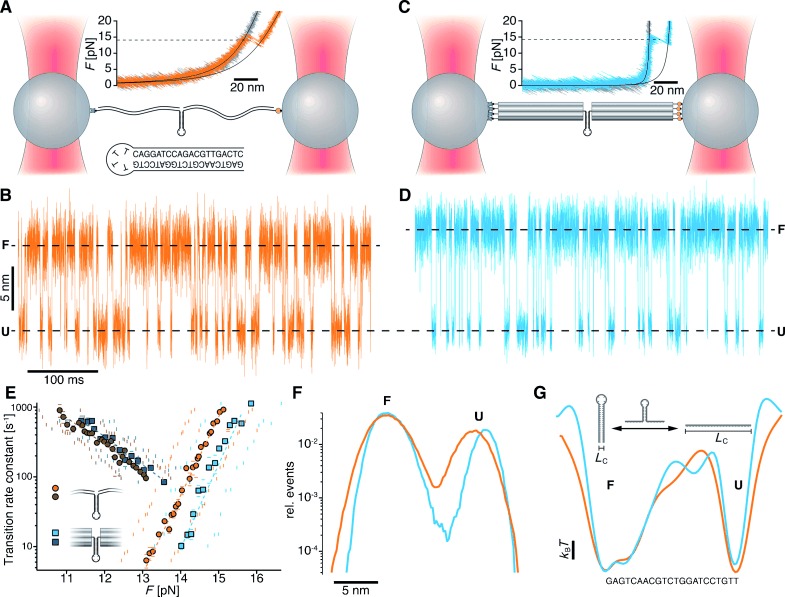
Single-molecule experiments with a stable 20 bp DNA hairpin. A,B) Conventional dsDNA linkers. A) Typical force-extension data at full measurement bandwidth. B) Typical extension data at full measurement bandwidth in constant trap distance experiments. F=folded; U=unfolded state of the hairpin. C,D) Same as in (A,B), but for experiments with ten-helix bundles. E) Force-dependent unfolding rate constants (rising branch) and refolding rate constants (falling branch) as obtained from dwell-time analysis in constant distance measurements (B,D) for different loads. The plot contains data from several molecules. Vertical and horizontal bars indicate errors of the rate constant determination. F) Histogram of the deflection states visited at a load of approximately 14 pN as measured with the conventional dsDNA (orange) versus the ten-helix bundles (cyan). G) Free-energy landscape reconstructed from (F) using deconvolution^22^ of the deflection statistics.

Finally, we used the stiff linker system to study equilibrium unfolding and refolding transitions of a weak six bp DNA hairpin. The force-extension response when pulling on constructs in which the six bp hairpin was either flanked by stiff linkers or the conventional dsDNA linkers featured, in both cases, increased extension fluctuations in the force range 4–8 pN (Figure 4 A–C), consistent with previous data for this hairpin.13a When monitoring the extension at a constant trap distance in this force range, the data collected with the stiff linkers exhibited two-state hopping signatures on the timescale of milliseconds that reflected reversible folding and unfolding transitions of the short hairpin (Figure 4 D). Such transitions could not be discerned in the data that we collected with the conventional dsDNA linkers (Figure 4 E). Hidden Markov modeling^14^ assuming a two-state system was successful when applied to the data acquired with the stiff linkers (Figure 4 D) and allowed for extraction of the force-dependent transition rate constants for the short hairpin (Figure 4 F). The corresponding analysis failed when applied to the data obtained with the conventional dsDNA linkers (Figure 4 E) because of the greater noise amplitude. Histograms of the extension signals obtained with the stiff linker system show two distinct populations that are separated by approximately 3 nm along the extension axis (Figure 4 G). These two populations are masked in noise for the case of the conventional linkers (Figure 4 H). The higher-resolution data acquired with the stiff linker system thus allowed construction of a meaningful free-energy landscape for the short hairpin that contains two minima (Figure 4 I).

**Figure 4:**
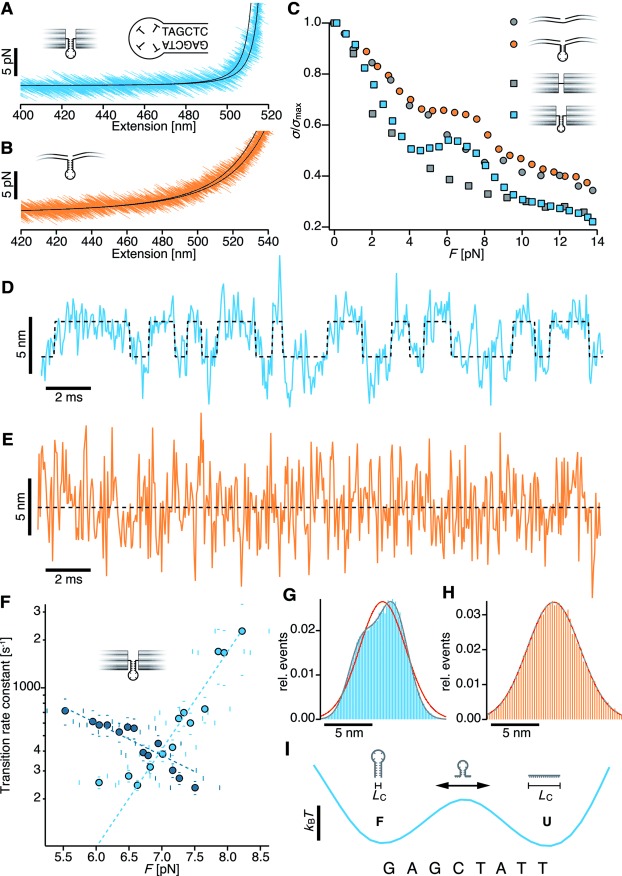
Conformational dynamics of a 6 bp DNA hairpin. A,B) Force-extension data at full measurement bandwidth collected with A) ten-helix bundles or B) conventional dsDNA linkers. C) Fluctuation amplitude in the data from (A,B) or when the linkers were connected directly (gray). D) Solid line: extension data for constant trap distance at full measurement bandwidth for the ten-helix bundles. Dashed line: fitted two-state hidden Markov transition trajectory. E) As in (D) but with conventional dsDNA. F) Force-dependent folding (falling branch) and unfolding (rising branch) rate constants as determined from dwell-time analysis of constant distance data as in (D) acquired at different loads. Vertical and horizontal bars indicate errors. G,H) Histogram of the deflection states visited by the system at constant trap distance for G) the ten-helix bundles or H) conventional dsDNA. Solid lines: fits using a single Gaussian (red) or a linear combination of two Gaussians (gray). The average force load on the folded and unfolded states was 6.5 pN and 6.0 pN in (G), respectively. I) Free-energy landscape reconstructed from the data in (G).

Our results thus establish DNA helix bundles as an attractive linker system for single-molecule mechanical assays. In addition to enabling single-molecule force spectroscopy with higher resolution in the low force regime, the system provides a multivalent and thus presumably longer-lasting attachment to the beads. It may also be conjugated to a wide range of target molecules using previously established methods.^3^ We speculate that also reducing the flexibility of the bead attachment may further enhance the performance of the stiff linker system. For the future studies of the energy landscape of enzymes and other functional protein systems, where conformational changes take place in the sub-nanometer range, these rigid DNA beams offer significant advantages over conventional approaches. Hence, we anticipate that our stiff linker system, based on self-assembled DNA nanostructures, may become a standard technique for the study of the functionally relevant dynamics of biological macromolecules.

## Experimental Section

DNA-templated design and synthesis: The multi-helix bundles were designed using caDNAno v 0.2.^15^ DNA scaffold strands were prepared as previously described.^16^ DNA staple oligonucleotide strands (Table S1) were prepared by solid-phase chemical synthesis (Eurofins MWG) with Eurofins MWG high purity salt free purification grade. The objects were synthesized in a one-pot mixture containing 20 nm of a 7560 base long M13mp18-phage-derived genomic DNA, 200 nm oligonucleotide staples in a pH 8 buffer that included 5 mm Tris⋅base, 1 mm EDTA, 20 mm MgCl2, and 5 mm NaCl. The mixture was incubated at 65 °C for 15 min, then annealed from 60 °C to 43 °C over the course of 16 h, and then stored at 4 °C. Analysis of the reaction products by agarose gel electrophoresis (Figure S2) showed that the helix bundles assembled with acceptable yield.

Monte-Carlo simulations for Figure 1: The combined free-energy function for two tethered beads held in an optical trap was constructed by considering 3D harmonic potentials for the laser traps with a curvature of 0.4 pN nm^−1^ in the directions perpendicular to the laser beam, and 0.04 pN nm^−1^ along the beam direction, plus the expected energetic contributions of the tether as a function of its extension. To model the elasticity of the tether, the extensible worm-like chain (eWLC) model was used in the case of the conventional dsDNA linkers (persistence length *p*=50 nm, contour length=530 nm, and stretch modulus *K*=1 nN), while an extensible freely-jointed chain with two elements [Eq. (1)] was used to model the elasticity of the rigid ten-helix bundles with *K*=10 nN and *L*=485 nm.


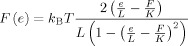
(1)

To account for the additional elasticity of the DNA single strands that were used to connect the stiff helix bundles to the beads, the worm-like chain model was employed using a contour length *L*=15 nm and a persistence length *p*=1 nm. The stochastic dynamics of the system were simulated using a Monte Carlo method.^17^ Every degree of freedom was varied randomly and simultaneously in discrete steps *n*. The difference in free energy with respect to the previous iteration was computed. The variations were accepted with Boltzmann-weighted probability. If the step was accepted, one trap position was moved away by d*x*=(500 nm s^−1^)/(100 kHz). If the step was not accepted, another random variation was performed. The resulting force-extension traces were downsampled to 20 kHz to maintain comparability to experimental data. The standard deviation of the extension signal versus force was determined in the same way as for the experimental data. For the standard-deviation plots shown in Figure 1 C, 200 individual force-extension traces were simulated and the average force-dependent standard deviation was determined.

Preparation of conventional dsDNA linkers conjugated to DNA hairpins: Autosticky-PCR^18^ was performed on a segment of M13mp18 genomic DNA to produce two products: 1) 5′ biotin+1024 bp+abasic site+hairpin+30nt 5′ overhang, and 2) 30nt 5′ overhang (complementary to 5′ overhang in product (1))+563 bp+5′ digoxigenin. The two products (1) and (2) were each purified by agarose gel electrophoresis followed by physical extraction using freeze’n’squeeze columns (Biorad), and then incubated together in a 1:1 volumetric ratio for dimerization. The dimerized product was again gel purified as above and thus gave an approximately 530 nm long dsDNA construct that included the hairpin at about 2/3 of its length.

Preparation of ten-helix bundles with hairpins: The bundles were self-assembled as described above. One version was prepared that included four biotinylated-DNA overhangs on one helical-bundle interface plus the desired hairpin sequence (extended by a single-stranded DNA overhang) on the opposing helical-bundle interface (see Figure S6). Another version was prepared that included four digoxigenin-modified DNA overhangs on one helical bundle interface and a single-stranded DNA overhang on the opposing interface that was complementary to the 5′ single-stranded overhang of the hairpin on the other ten-helix bundle. These bundles were gel-purified (2 % agarose, 0.5xTBE+11 mm MgCl_2_) followed by physical extraction. The samples were concentrated from 400 μL starting volumes to 20 μL final volume using 100 kDa molecular weight cutoff filters (Amicon, Millipore). The purified and concentrated bundles were incubated in a 1:1 volumetric ratio overnight at room temperature to induce dimerization through hybridization of the 5′ sticky ends of the hairpin. The dimerized products were again gel-purified followed by physical extraction.

Single-molecule laser-tweezer measurements: Purified sample solutions (1–4 μL) were mixed with 1 μL of streptavidin-labeled silica beads (Bang Labs, diluted 1:600) and NaCl (5 m, 1.9 μL) was added. Solutions were incubated at room temperature for about 1 h. Anti-digoxigenin-labeled silica beads (2 μL)^19^ and d-Glucose (3 μL, 5 % *v*/*v*, Sigma–Aldrich) were dissolved in 1×PBS, 2×PBS plus 400 mm NaCl (10.5 μL) was added and the solutions were vortexed. 3 μL of a solution containing 3.7 mg mL^−1^ glucose oxidase (Sigma–Aldrich) and 0.17 mg mL^−1^ catalase (Sigma–Aldrich) as in13a were added. Finally, the reaction volume was filled up to 30 μL using H_2_O, followed by mixing and transfer into the measurement chamber in a previously described self-built optical-trapping apparatus.^20^ All data were acquired at 30(±1)°C using a 100 kHz sampling rate and post-acquisition downsampled to 20 kHz. The data was hardware-filtered with a Butterworth filter with a cutoff frequency of 32.6 kHz. The trap stiffness was set to approximately 0.4 pN nm^−1^ in every measurement. Stretch-and-relax measurements were performed at 500 nm s^−1^ with a maximal force-load of about 30 pN. To calibrate the bead-deflection signals into actual bead displacements away from the trap center, a pair of beads was trapped and held at a distance of 10 μm and a previously described calibration procedure was used.^21^ 100 power spectra (each 125 ms) were recorded and averaged while moving the sample stage sinusoidally at a frequency of 32 Hz, which produces an additional peak in the power spectra. The averaged power spectra obtained for both traps were fit individually according to a previously described equation^21^ to determine the deflection sensitivity (nm V^−1^) and stiffness parameters (pN nm^−1^) for both traps. The voltage signals in the bead deflection signals for both traps were calibrated into bead displacements using the sensitivity parameters. Bead displacement signals were converted into forces using the stiffness parameters. A baseline was determined by acquiring a set of deflection data points while moving the beads towards each other and fitting the displacement signals with a polynomial of eighth grade individually for the two traps to account for beam crosstalk at short distances. The baselines were subtracted individually from the deflection signals obtained for both traps. After calibration, the part of the deflection time traces where the beads are brought into contact was analyzed for correlation. Pearson’s *r* correlation decreases from a value of 0 to approximately −0.5 when the beads physically touch each other. The first point after the decrease in correlation was taken as the zero deflection value. The standard deviation of extension versus force traces were obtained by computing the standard deviation of the extension signal as well as the average force load in a moving window of 200 data points width. The constant distance data was evaluated as previously described.^14^ For the deconvolution of deflection histograms, a previously described algorithm was used.^22^ The necessary force-dependent point-spread function was determined experimentally using control constructs that lacked a DNA hairpin. In energy landscapes, the deflection axis was transformed first into contour length space using elastic parameters from the eWLC/WLC fits to the force-extension data and slightly offset (±5 nm) to shift the small-contour length minimum in the energy landscape to zero contour length, and then mapped onto the sequence along the hairpin stem.

## Supplementary material

As a service to our authors and readers, this journal provides supporting information supplied by the authors. Such materials are peer reviewed and may be re-organized for online delivery, but are not copy-edited or typeset. Technical support issues arising from supporting information (other than missing files) should be addressed to the authors.
