# Supplementary material for: Rigid DNA Beams for High-Resolution Single-Molecule Mechanics
Source: Angew Chem Int Ed Engl. 2013 Jun 21;52(30):7766–71. doi: 10.1002/anie.201302727 (PMC3749440; doi:10.1002/anie.201302727)
Supplement: Supplementary file 1 [file anie0052-7766-SD1.pdf]

Angewandte Chemie
Eine Zeitschrift der Gesellschaft Deutscher Chemiker

Supporting Information

© Wiley-VCH 2013

69451 Weinheim, Germany

Rigid DNA Beams for High-Resolution Single-Molecule Mechanics**

*Emanuel Pfitzner, Christian Wachauf, Fabian Kilchherr, Benjamin Pelz, William M. Shih, Matthias Rief, and Hendrik Dietz**

anie_201302727_sm_miscellaneous_information.pdf

Figure S1

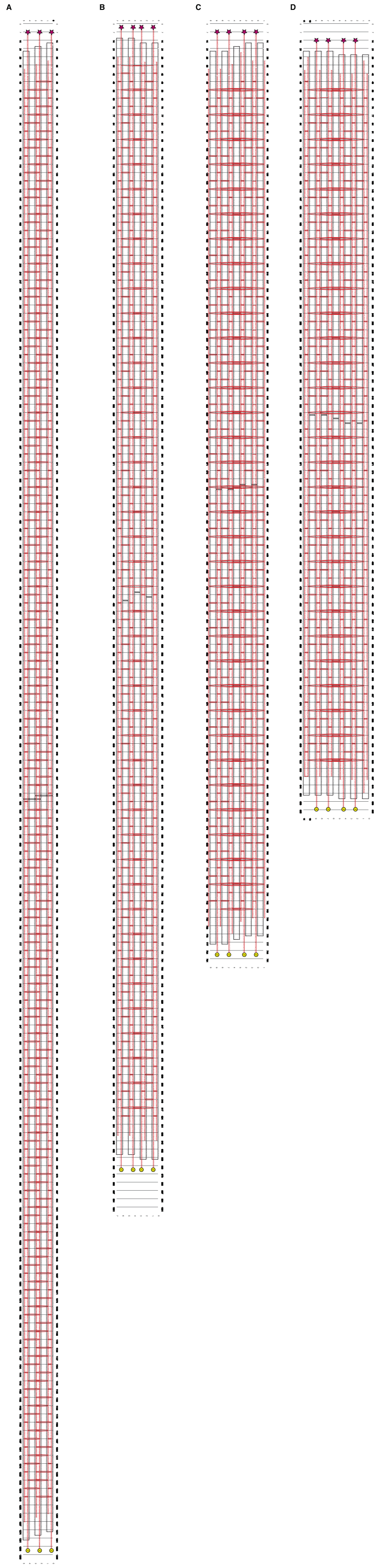

Fig. S1: caDNAno design diagrams. Scaffold strand path is depicted with black lines, and staple strand paths are shown as red lines.

Figure S2

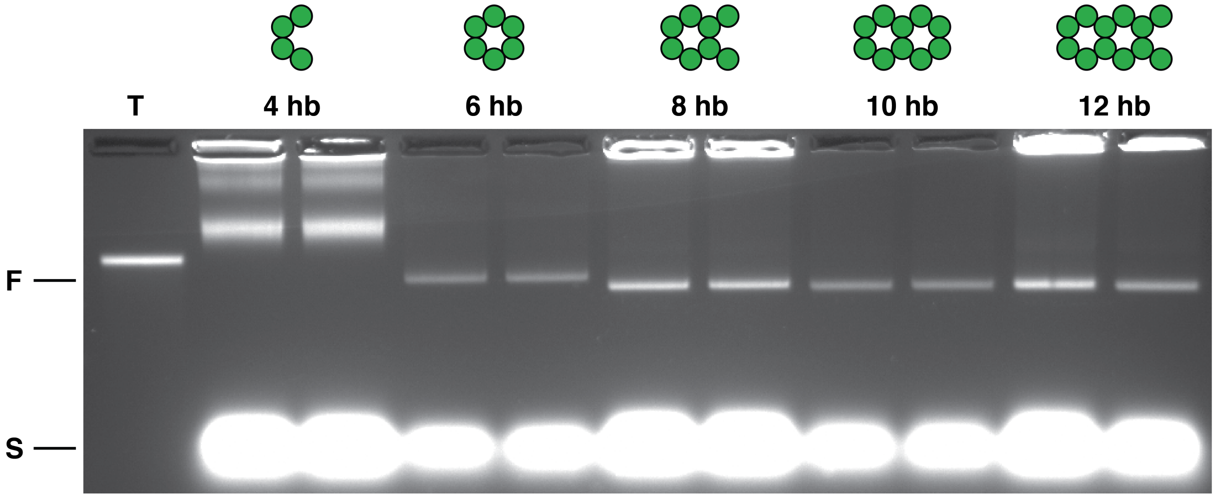

Fig. S2: Gel-electrophoretic bundle folding quality assessment. Photograph of a 2% ethidium-bromide stained 2% agarose gel on which a reaction products were electrophoresed. The gel also includes a four helix bundle not described in the main text. The low electrophoretic mobility of the folded species as compared to the other bundle types is due to the high flexibility (random-coil-like) of this bundle type. The four helix bundle was thus not considered for usage as a linker system. "T" marks the lane on which the template strand was electrophoresed. "F" marks the folded species, "S" excess staple strands.

Figure S3

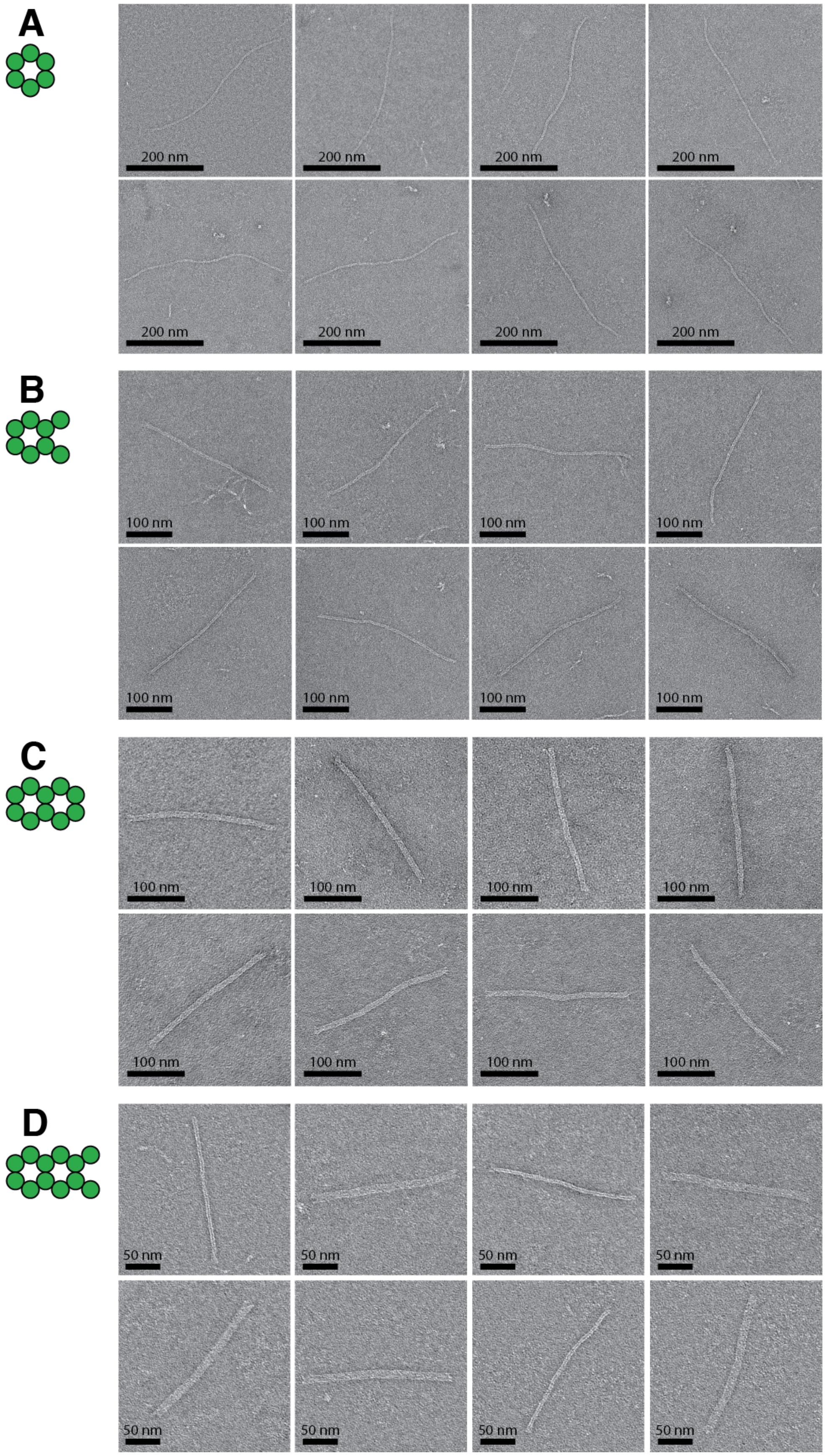

Fig. S3: Exemplary negative-stain transmission electron micrographs of purified DNA helix bundle particles.

Figure S4

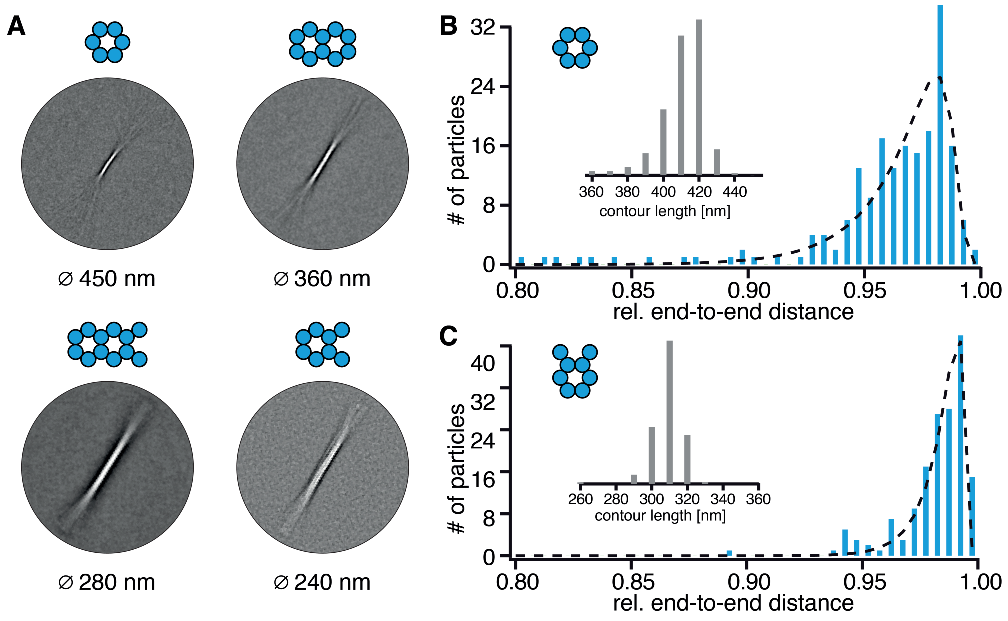

Fig. S4: (A) Average TEM-micrographs obtained by rotational alignment of single particle micrographs of each bundle type against a randomly chosen reference micrograph. (B,C) Blue Bars: Histograms of the relative end-to-end distance observed for individual six (B) and eight (C) helix bundle particles. The absolute contour lengths (inset, grey bars) were determined by manual backbone tracing, where the two endpoints of a backbone trace were used to determine the absolute end-to-end distance. Dashed lines: fit by a semi-flexible beam model. [10]

Figure S5

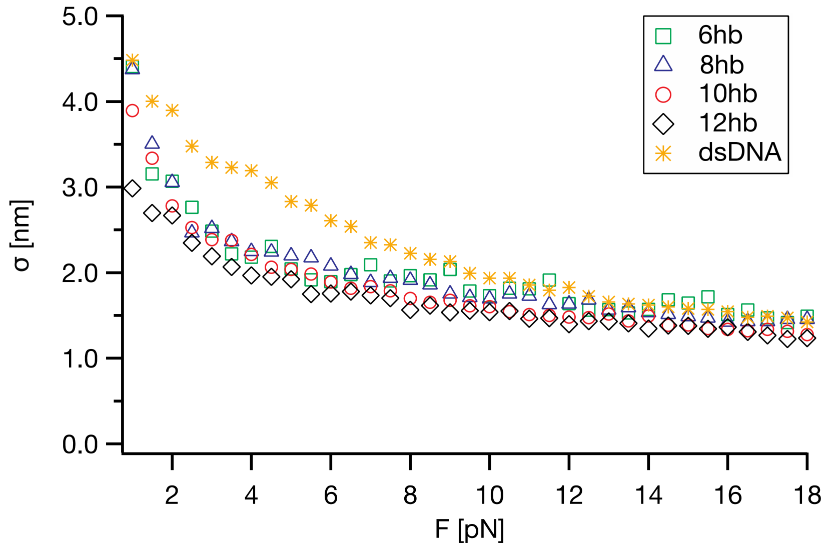

Fig. S5: Force-dependent noise-amplitudes for stiff DNA helix bundle linkers vs. conventional dsDNA linkers.

Figure S6

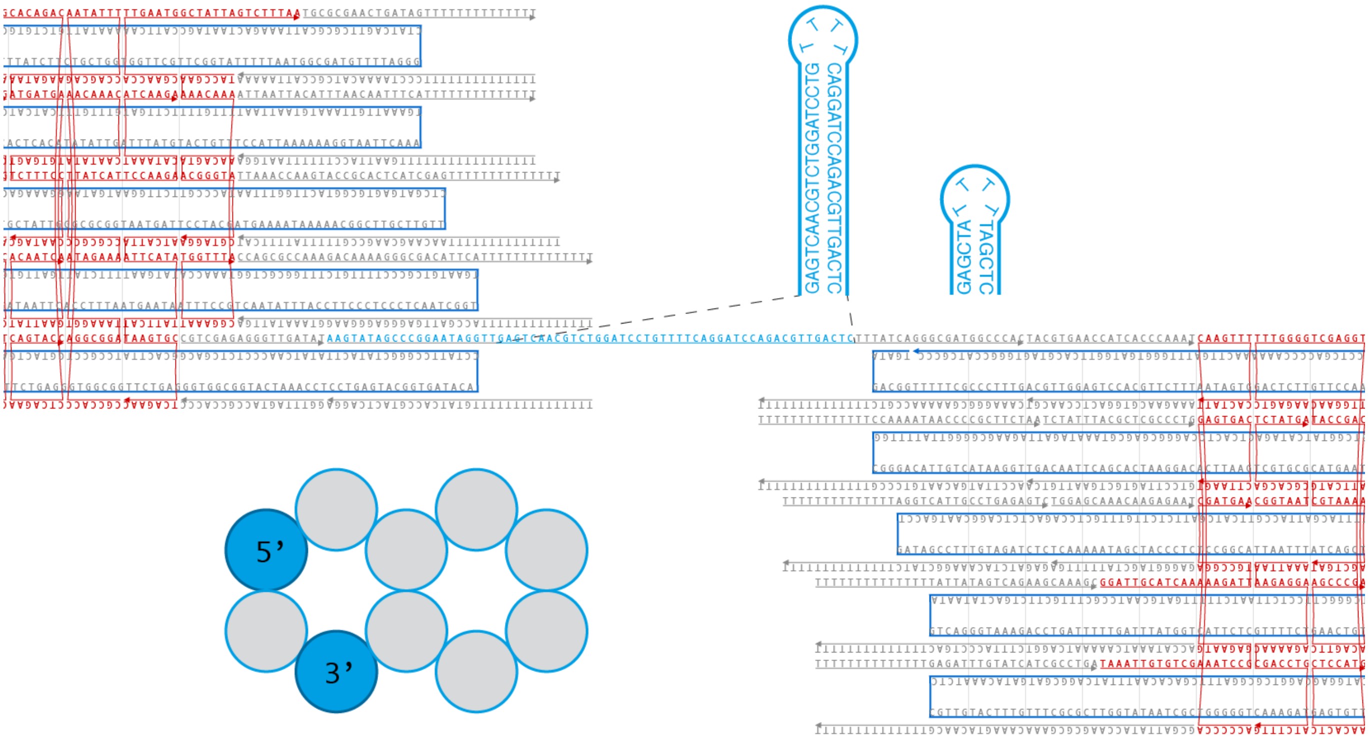

Fig. S6: Design diagram for hairpin-helix bundle constructs used for the experiments described in figures 3 and 4.

Figure S7

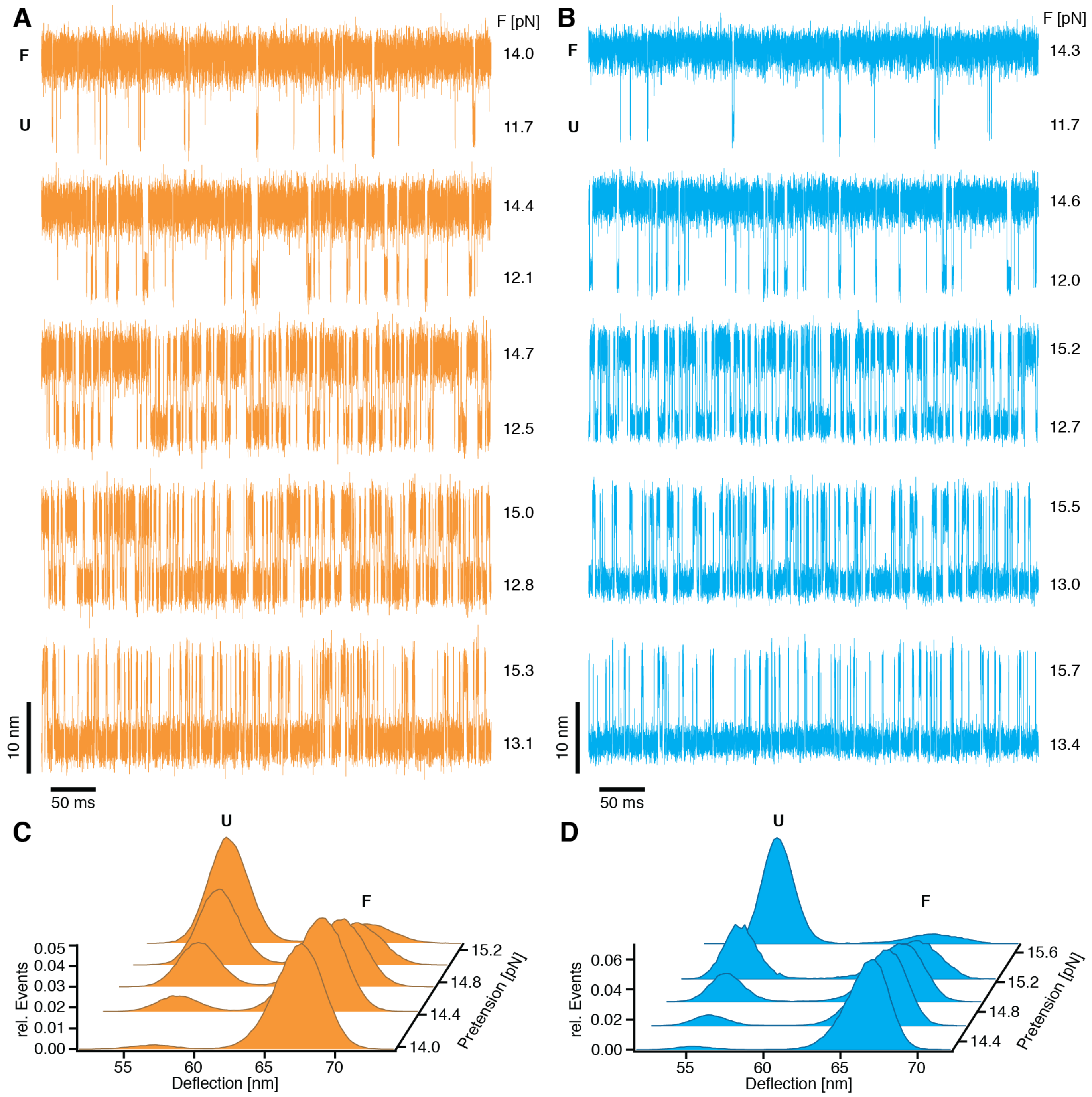

Fig. S7: Additional constant-distance data as in Fig. 3 obtained for different loads. Orange traces: data collected with conventional linker system. Cyan traces: data collected with stiff ten helix bundles.

Figure S8

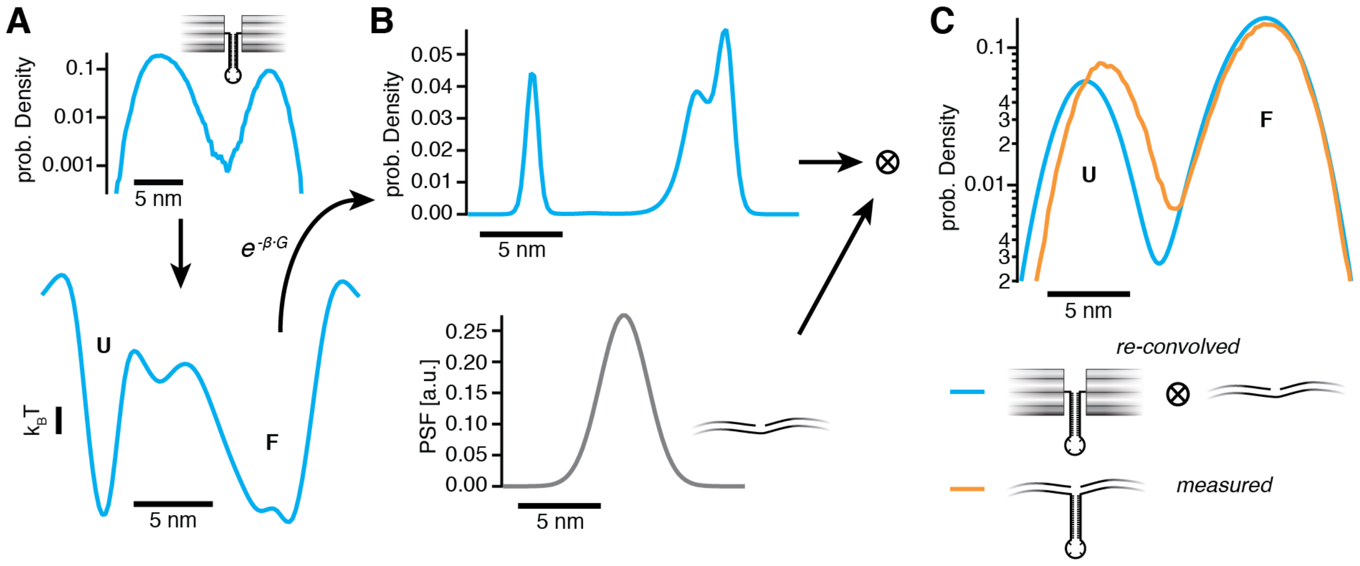

Fig. S8: Schematic explanation of the workflow for deconvolution of a deflection histogram to extract the energy landscape for the 20-bp DNA hairpin from figure 3, followed by reconvolution using the point-spread-function of the conventional dsDNA handle system.

Sequence Information

6 helix bundle

Start	End	Sequence	Length
0[370]	4[357]	CCCGCTTATTAAGTGATTCAAAAGGGTGTAAGAGGCCAACTT	42
0[412]	4[399]	TAATGAGTAAAACGTCATATATTTAAAGAGCTTCGGAACGA	42
0[454]	4[441]	AACATACGTGGATGGAAGCCTTTATTTCCAGGATTAAATTGT	42
0[496]	4[483]	CTGTTTCTAAGTGTGTTGTACCAAAAACTCATTTTGCGCGAA	42
0[538]	4[525]	GATCCCCCCGCCAAGCAAAATTAAGCAAGCTGTAGTACACTA	42
0[580]	4[567]	GGCTTAAGGAGTGAGCATTAACATCCAAGTCTGGACCACTAC	42
0[622]	4[609]	TCTCCGAAAGCAACGCGCGAGCTGAAAAGCGAACGTTTCATG	42
0[706]	4[693]	AATTTAAGCGTTATATTATTCATTAAAGTTAGCAATGCGGGA	42
0[748]	4[735]	ACTTTTTGTAGGGCACATTCAACCGATTGCACCGTATATTCG	42
0[790]	4[777]	ATGCTGATGTAATTAGAAAATTCATATGTCAGACTTGCGCCG	42
0[832]	4[819]	TTTTAACTATAAAGCGCAAAGACACCACCCCTTATTGCTTTC	42
0[874]	4[861]	CAATAGTCGACAATCGTAGAAAATACATGAACCAGAAGGCTC	42
0[916]	4[903]	TGAAAACTTATCAACTGGCATGATTAAGCCCTCAGATTGCGA	42
0[958]	4[945]	TCTGTAACATCCTAGGAAACCGAGGAAACCACCAGACAGTTT	42
1[385]	3[398]	TTTTTATACGGTCATCGCGTTTTAATTCTGCAATGCAGTCAC	42
1[427]	3[440]	TCACGCATCCGCGAAAACTCCAACAGGTAACGCAACAGGAGA	42
1[469]	3[482]	ATAACATACAACGGCTTTTGATAAGAGGATTATGATCATGCG	42
1[511]	3[524]	TTGCTGGCATCTTTTTGCTGAATATAATTAAAGCCCTTATGA	42
1[553]	3[566]	ACAGGAACCAACCTAACTAAAGTACGGTTAAATCATTTACGC	42
1[595]	3[608]	ATCGTCTTTCCATTTGATTCCCAATTCTGGTGGCAGCCCTGC	42
1[679]	3[692]	GAACCCTCCTCAGCTAGCACCATTACCAGTGAATTGTTTAGT	42
1[721]	3[734]	AATATTTAGGCTTGAACCATCGATAGCAGAGGGAGGCCAACG	42
1[763]	3[776]	AGCCCTACAACAACGTTTGCCTTTAGCGGTTTACCTAACAAC	42
1[805]	3[818]	GCAGAAGATTTCTTTTTCGGTCATAGCCGGAATAACAGTAAT	42
1[847]	3[860]	CCTGCAAAGCCTTTAATCAAAATCACCGACATAAATTCTGTC	42
1[889]	3[902]	TCTAAAGTTTTTTCCCTCAGAGCCGCCAACTCCTTCAGAACG	42
1[931]	3[944]	ATCAATAGTGAGAACACCCTCAGAGCCGCGCAATAGAAAAAT	42
2[384]	0[371]	AATGTGTTCAAATAATCATAAGGGAACCAATCCTGCTCACTG	42
2[426]	0[413]	AATTTTTCGGAAGCCCTGCTCCATGTTAAAAGAGTGGGTGCC	42
2[468]	0[455]	ATACTTTATTGCTCAGATTTGTATCATCTTCTTTGTCCACAC	42
2[510]	0[497]	ATAAAGCAGCTTAAGACCCCCAGCGATTTCAAACTGTCATAG	42
2[552]	0[539]	AAGGCAAAATATGCAAAACGAAAGAGGCCGCCAGCGACGGAG	42
2[594]	0[581]	TACTAATTAACAGTAAACGGGTAAAATACATTTTGTAAACAG	42
2[636]	0[623]	TATTTTCGACCATTGGCTTTGAGGACTACAGATTCAATCATT	42
2[720]	0[707]	AAATATTCCAATGACAGGGAGTTAAAGGTACGTGGTGACCTA	42
2[762]	0[749]	AAGACAAGAATCAACATCGCCCACGCATATGCGCGCGAGAAA	42
2[804]	0[791]	TTGTCACTCGGCATAAACAGCTTGATACCGAACGATATGTAA	42
2[846]	0[833]	ACATATATTTTCATAATTGTATCGGTTTCAGTATTACTACCT	42
2[888]	0[875]	AGTATGTCCGCCTCACGTTGAAAATCTCCAGCAAAGAAGAGT	42
2[930]	0[917]	AATACCCGAGCCACTAGAAAGGAACAACATATCAAGAATCCT	42
3[357]	5[370]	CAAGGCGTCCAGTCGAACGGTACGCCAGGAACTGAAAGCCCG	42
3[399]	5[412]	GACGTTGTGAGCTAGAGGCCACCGAGTACTTAGCCAAAGCGA	42
3[441]	5[454]	AGCCAGGGAGCCGGCGTTGTAGCAATACGCCTGATAGAGAGT	42
3[483]	5[496]	CACGACTCTGTGTGCTGAGTAGAAGAACATACCAATGCGGAT	42
3[525]	5[538]	CAATGTCGGGTACCCAGAACAATATTACAAAAGAACTCAACA	42
3[567]	5[580]	TCGCCCTGCTACGTCATGGAAATACCTACGTAATGAGTTTCA	42
3[609]	5[622]	CATCTGTACTCTGAATTATTTACATTGGAAGACTTAGTAGAT	42
3[693]	5[706]	ATCATATTGGTTTGTGAAAGCGTAAGAACCGCTTTGGCCGGA	42
3[735]	5[748]	CTCAACACAAATATGCTATTAGTCTTTAAACCGATAATCAGT	42
3[777]	5[790]	GCCAACATGCAAATGCCATTAAAAATACCGATAGTGTAGCGC	42
3[819]	5[832]	AAGAGAACTCCGGCAGAGGTGAGGCGGTATCAGCTTAGCGTT	42
3[861]	5[874]	CAGACGAGAATTTAACGCTGAGAGCCAGCAAAAAAAGCCACC	42
3[903]	5[916]	CGCCTGTATAGCGATTGCTGAACCTCAATAAAGGAAACCGCC	42
3[945]	5[958]	AATATCCATCGTCGAGTTGGCAAATCAAACTTTCAAACCACC	42
4[398]	2[385]	GGCGCAGAATCAGTACTCACATTAATTGGTTTTCCCCTGAGT	42
4[440]	2[427]	GTCGAAAAATTAACAAGCATAAAGTGTAGCTTTCTGGATAAA	42
4[482]	2[469]	ACAAAGTCACTTGCAAATTGTTATCCGCTGTGAATCCCTGTA	42
4[524]	2[511]	AAACACTTAATATCGAGCTCGAATTCGTTGTCAACTCAGAGC	42
4[566]	2[553]	GAAGGCAAAACGCTGGTGCTTGTTACCTTAATCTATACAGGC	42
4[608]	2[595]	AGGAAGTGAAATGGCCTCCTGGTTGGTGCAGTGCGTCAATTC	42
4[692]	2[679]	TCGTCACTCTGACCAAATACCGACCGTGAAAGCCTATCACCG	42
4[734]	2[721]	GTCGCTGTTGAATGATTTTAGTTAATTTGTATAAAGGAAGGT	42
4[776]	2[763]	ACAATGAAAACATCCCAATCGCAAGACACCATATTAGCGCCA	42
4[818]	2[805]	GAGGTGAATAAAACTTAGGTTGGGTTATTTCGAGCGTTTATT	42
4[860]	2[847]	CAAAAGGCAGTGCCTCAAAATCATAGGTAAAGTAAGGTGGCA	42
4[902]	2[889]	ATAATAACATCACCTAGCTTAGATTAAGGCTAATGATTACGC	42
4[944]	2[931]	CAGCGGATCTGGTCCTATTAATTAATTTGAACAAATAACGG	42
5[371]	1[384]	AAAGACTAGGTAAATGGGTAACGCCAGGCGTTGCGAGAAGTG	42
5[413]	1[426]	ACCAGACAGAACCCACGGCCAGTGCCAAAAGCCTGCTGTCCA	42
5[455]	1[468]	ACCTTTATGCGGGATTCTTCTAAGTGGTTCACAATATTAGTA	42
5[497]	1[510]	GGCTTAGTAAATCGCCTTAGTGCTGAATAATCATGATCGGCC	42
5[539]	1[552]	TGTTTTAAGAATTAAATAACCCCGCTTCCGATAAACATTGCA	42
5[581]	1[594]	TTCCATAAGTAGTACTCTATGATACCGATAATGAGACGCTCA	42
5[623]	1[636]	TTAGTTTATTTGGGTCGTCGGTGGGCACTCACATAACCAGTC	42
5[707]	1[720]	AACGTCAGACGGAAACAAATTCTTACCACATCTTCCACAGAC	42
5[749]	1[762]	AGCGACAAAGGGCGTTAATTGAGAATCGAAGAACGAACTGAT	42
5[791]	1[804]	GTTTTCAAATCAATTAGGCAGAGGCATTATAACTAACCACCA	42
5[833]	1[846]	TGCCATCAAAGAAATACCGACAAAAGGTCTGAGAGAACACCG	42
5[875]	1[888]	ACCGGAATAGCAAAAAACAACATGTTCAACGCTGATGAAAAA	42
5[917]	1[930]	ACCCTCAAAAAGAACAATAGATAAGTCCTCCCTTAACCCTCA	42
0[664]	5[664]	TTAAATAAGAATAAAAAAGGGACATTCTTCGGAACTAGAGCC	42
1[637]	4[651]	ACACGACCTACAGAAGATACATTTCGCAAATGGATGAGGGTA	42
2[678]	0[665]	TCACCGATCACCAGAGCGAAAGACAGCAGGCCAACTAAGGCG	42
3[658]	3[657]	CCGGAATCATTTGGGATCAATAACCTGTGGGGCCTTGAAACA	42
4[650]	2[637]	GCAACGGCAGTAATTCGGCTGACGCATTGAATATATTAGCTA	42
5[665]	1[678]	AGCAAAACTTGAGCCATAATTACTAGAATGATAAAAGAGATA	42
0[76]	4[63]	AAGAGTCGTAACAATTAAATTTTTGTTAGCCAAAAGAACTAA	42
0[118]	4[105]	GAATAGCGACCGTAAATATTTAAATTGTAACCCTCACCAGTC	42
0[160]	4[147]	GATGGTGGTAACCGGATAATCAGAAAAGAGGCTTTATTGTGA	42
0[202]	4[189]	GCGGTCCGGCCTCAGGTAATCGTAAAACAATGTTTGTAGTAA	42
0[244]	4[231]	CTGATTGTTCTGGTTCATTGCCTGAGAGAAATATTTTCAGTG	42
0[286]	4[273]	CGCCAGGGCTGCGCGAGAGGGTAGCTATAAAACGAACCGGAT	42
0[328]	4[315]	TGAATCGGCTATTATCAATATGATATTCGACTATTGCATAGG	42
0[1000]	4[987]	AAACAGTTGTCTTTTTTTAAGAAAAGTAGGTTGAGGTAAATG	42
0[1042]	4[1029]	AATTACACCGCACTCAAGAAACAATGAAAATAAATTTAGCGT	42
0[1084]	4[1071]	AGCAAAATAGGAATAGAGATAACCCACAAATTTACCAAACTA	42
0[1126]	4[1113]	TACAAAAGAAGGCTCACCCTGAACAAAGACAGGAGATAGGAA	42
0[1168]	4[1155]	AGAAACAAACCTCCCATAAAAACAGGGATGAGTAACCCTCAG	42
0[1210]	4[1197]	AACGTCATTAGTTGTAACGTCAAAAATGTTTCGGATTAGTAC	42
0[1252]	3[1263]	ACGTAAATTACCAACGCTAACGAGC	25
1[31]	4[44]	GTGAACCATCACCCAAATCAAGTTTACATTATTACAG	37
1[91]	3[104]	ACCCTAATGGGAAGAGCAACACTATCATAAACGTTACAAACG	42
1[133]	3[146]	CGGCGAATATGCGAACCAAAATAGCGAGCCCCAAATAGATGG	42
1[175]	3[188]	CGGGCGCTTGAGATGGGGGTAATAGTAATAGCATGACGACGA	42
1[217]	3[230]	TAACCACCTTGCCCTGCGGAATCGTCATTCTGGAGCTTTCCG	42
1[259]	3[272]	CGTACTAACCCAAATTTAAACAGTTCAGTTTTGAGCATTCGC	42
1[301]	3[314]	TCGTTAGACCTTCACAGGTCTTTACCCTAACCGTTGTGCGGG	42
1[343]	3[356]	GGGATTTGGACAGATGCATCAAAAAGATAGAAAGGTGTGCTG	42
1[973]	3[986]	TGAGGAATGTATGGCAGCATTGACAGGAAGCAGATAATCAAT	42
1[1015]	3[1028]	AATAGATTAAAGTTGATATTCACAAACAATAGCAATATTAAA	42
1[1057]	3[1070]	GAAGTATTGTAGCAAAGCGCAGTCTCTGAGAATTGTTTTATT	42
1[1099]	3[1112]	TCCTTTGACCGTAATGGCTTTTGATGATTCAGAGGGCAAATC	42
1[1141]	3[1154]	TTATCATACCCTCACGGGGTCAGTGCCTAGCGCATGAGGCGT	42
1[1183]	3[1196]	TTATCATCTCAGAAATGCCCCCTGCCTAAAAATAGGCCTTAA	42
1[1225]	3[1238]	ATATAATAATAGGTAAGTATTAAGAGGCCAATCCATTTTATC	42
2[90]	0[77]	TGTTAAATAGTAAGAAAAATCTACGTTAAGCACTATTGGAAC	42
2[132]	0[119]	AGATTGTGATAAAATTTTAAGAACTGGCTTGACGGATCAAAA	42
2[174]	0[161]	TATGTACTGCCAGAGGTTTAATTTCAACGAAAGCGCCTGTTT	42
2[216]	0[203]	GAGAATCCCAATACTGACGAGAAACACCGGTCACGGCAGCAA	42
2[258]	0[245]	CAAAGGCCAAATGCTCAACGTAACAAAGGCCGCTAGCAACAG	42
2[300]	0[287]	ATAAATTCAAAAATTCAAGAGTAATCTTATAACGTTATTGGG	42
2[342]	0[329]	CAGTCAAAGCGGATTGAACGGTGTACAGGGAGGCCGCATTAA	42
2[972]	0[959]	CAAAGTTCCGCCGCGATTTTGCTAAACACAGTTGACCTTGCT	42
2[1014]	0[1001]	CTTACCGTGGCCTTTTGTCGTCTTTCCAGAGCACTTTAATGG	42
2[1056]	0[1043]	CCCAATAGAATGGATTCCACAGACAGCCCATTTGACAAAATT	42
2[1098]	0[1085]	AGCGCTATCATACACACTGAGTTTCGTCCAACTCGTTACCTG	42
2[1140]	0[1127]	GAGAATTGTTTTAATTTTCAGGGATAGCAAGTTTGACCAAGT	42
2[1182]	0[1169]	TACAGAGACAGTTACCGCCACCCTCAGACAGAAGGCATCGGG	42
2[1224]	0[1211]	AACGATTAACATGAGTATCACCGTACTCATGGCAACAGGTTT	42
2[1252]	4[1239]	CATATTATTTATCCTGAGACTTAAGTAT	28
3[34]	0[37]	CTGTAGCCAGCTTTCCCAACGTCAAAG	27
3[63]	5[76]	TGAGCGACACTATTTCGAGGTGCCGTAAATAAAACGGAATTA	42
3[105]	5[118]	GCGGATTCCGAGATCCCCGATTTAGAGCTCATTATGTTTACC	42
3[147]	5[160]	GCGCATCGTTCCGAAGAAAGGAAGGGAATTTAATCTGCAAAA	42
3[189]	5[202]	CAGTATCACGCTGGCTGGCAAGTGTAGCAGAACGAAGACTGG	42
3[231]	5[244]	GCACCGCCCCTTCAGCCGCGCTTAATGCCTGCTCACATTGAA	42
3[273]	5[286]	CATTCAGGTGGTTTTTTGACGAGCACGTGACAAGAGAATGAC	42
3[315]	5[328]	CCTCTTCGCCAACGGCGGGAGCTAAACAACCAGGCATAGTCA	42
3[987]	5[1000]	AATCGGCACATAAATAAAATATCTTTAGGACGTTAGCAGGTC	42
3[1029]	5[1042]	CCAAGTATTTAACAGTCAATAGATAATACTCATAGCCTCATT	42
3[1071]	5[1084]	TTCATCGGAAGATGTACAAACAATTCGAACCAGTACGTTCCA	42
3[1113]	5[1126]	AGATATATCGCGCAGTTATTAATTTTAAAAGCCCATGTACTG	42
3[1155]	5[1168]	TTTAGCGATAACGGAACAAAGAAACCACACCGCCACAGTGCC	42
3[1197]	5[1210]	ATCAAGAGATGAATCTGATTATCAGATGAGGAGGTACCTATT	42
3[1239]	1[1260]	CTGAATCACAGAAAGTTTGGATTATACTTCTGAATA	36
4[62]	3[62]	CGGAACATTTGGGGAAAGAACGTGGACTATCAACATTAAATG	42
4[104]	2[91]	AGGACGTAGGGAGCAGGGTTGAGTGTTGCGTGGGAAATATTT	42
4[146]	2[133]	ATTACCTCGTGGCGAATCGGCAAAATCCGTTGGTGAACAGGA	42
4[188]	2[175]	ATTGGGCTAGGGCGTTTGCCCCAGCAGGTGAGGGGTCAATCA	42
4[230]	2[217]	AATAAGGCACACCCCCGCCTGGCCCTGACAGCCAGCAAACAA	42
4[272]	2[259]	ATTCATTTGGTTGCTTCTTTTCACCAGTAAAGCGCAGATCTA	42
4[314]	2[301]	CTGGCTGAATCAGACGCGGGGAGAGGCGGCGATCGCTAGCTG	42
4[356]	2[343]	TGAAAGATAGACAGGGGAAACCTGTCGTAGGGGGACCGGAGA	42
4[986]	2[973]	AATTTTCGGTTATCTCAATATATGTGAGAGAAACCAGCCGAA	42
4[1028]	2[1015]	AACGATCTAGAGCCATTTCATTTGAATTGAACGGGTAGCTAT	42
4[1070]	2[1057]	CAACGCCTAGACTTATGAAACAAACATCAAGCCGTAGTTAAG	42
4[1112]	2[1099]	CCCATGTCCCGAACGAGGCGAATTATTCATAGCAAGTAATTG	42
4[1154]	2[1141]	AGCCACCTTTGCGGATTCGCCTGATTGCAGAACGCTAGACGG	42
4[1196]	2[1183]	CGCCACCCATATTCATACAGTAACAGTATTTTGAACAGCCTT	42
4[1238]	2[1225]	AGCCCGGCCTGATTTAAAGAAATTGCGTGCTACAAAATAAGA	42
4[1259]	5[1267]	CGAGAGGGTTGATACCTCAAGAGAAGGATTAGGATT	36
5[38]	2[49]	CATTCAACTAATGCAGATACATAACAATCAGCTCATTTT	39
5[77]	1[90]	CGAGGCAATTCGCACCCGTCGGATTCTCTTCCAGTAATCGGA	42
5[119]	1[132]	AGACGACATAAGCAATGGGATAGGTCACCTTATAAGGAAAGC	42
5[161]	1[174]	GAAGTTTCCCGGTTTGCATCTGCCAGTTCGAAAATAAAGGAG	42
5[203]	1[216]	ATAGCGTGATGAACGGAAGATCGCACTCGAGAGTTCTGCGCG	42
5[245]	1[258]	TCCCCCTTATCAGGGCCGGAAACCAGGCGAGACGGCAGGGCG	42
5[287]	1[300]	CATAAATAATGCCGAACTGTTGGGAAGGGTTTGCGGCTTTCC	42
5[329]	1[342]	GAAGCAAATCACCACGCCAGCTGGCGAAGCCAGCTGATTAAA	42
5[959]	1[972]	ACCAGAGACCAGAAATTTACGAGCATGTTGAATAAAAGGAAT	42
5[1001]	1[1014]	AGACGATAAGCCCTCCTTATCATTCCAAACCTTTTAACAACT	42
5[1043]	1[1056]	AAAGCCAATAAGAGCATCGAGAACAAGCAAGAAAAGGATTTA	42
5[1085]	1[1098]	GTAAGCGATATCAGCATTACCGCGCCCAATTTCAATATTAAA	42
5[1127]	1[1140]	GTAATAAAACTGAATATCCGGTATTCTATTTGAATAGTAACA	42
5[1169]	1[1182]	CGTATAAAGAATAACGACTTGCGGGAGGCCTTTTAAGCGGAA	42
5[1211]	1[1224]	ATTCTGATTTTGTTCTATTTTGCACCCAAGATTTTTCATCA	42

biotin-modified
GTAGAAAGATTCATCAGTTGAGGGTGGTGGTTGTT - Bio
TTAACCAATAGGAACGCCATCAAAAATAATGTGTTGGTGT - Bio
GGCGAAAAACCGTCTATCAGGGTTGGGT -Bio

digoxigenin-modified
Dig - TTTTGTGAGTACCAGGCGGATAAGTGCCGT
Dig - TGTGGTTTGTGAATTTGCCAGTTACAAAATAAACAGC
Dig - TTTGTTGTGGTGGGCCTACCATATCAAAATTATTTGC

8 helix bundle

Start	End	Sequence	Length
0[48]	5[55]	ACGTCAAAAATCAAACCCTCATATCTAAAATATCTTTTAATGGCCTGTT	49
0[468]	2[476]	CAATTCCAGTAATAATCGGCCCTGATTGCTTTGAATACCAATGAAGAAC	49
0[489]	7[503]	TGTGAAAAGCGGTTGATAATCTTCTTAACGTTTGCCATCTTT	42
0[909]	5[916]	GACGTTGTGTTGGGAATAGTAAACGAGTAGATTTAATCTTGAAGGGAAC	49
0[944]	1[944]	ATTAAGTTGGGTAACGCCAGGCGGGCCTCTTCGCTATTACGC	42
2[55]	7[62]	ATATCAAGTTTTTTCCCTTTTAAAAGAACTGGCAT	35
2[475]	7[482]	TCAAACTACATCACGTATTCTCGGTCATAGCCCCC	35
2[496]	5[496]	TCATATGTACCCTAGTTTAGACTGGATAAGGGGGTCTTGATA	42
2[916]	7[916]	TTCTACTAAGGGCGTAGGTGTCAAGAGA	28
2[944]	3[937]	GGGGCGCGAGCTGAAAAGGTGCATTAGATACATTT	35
4[41]	2[32]	ATACCGAGGAAGGTATCAATATCT	24
4[930]	2[917]	CTTCATCAAGAGTAGTTTGACGCATCAA	28
5[28]	0[32]	GAATCATAATTACTAGTAAGCATCACCCAGGGCGAAAA	38
5[56]	0[49]	TAGTATCACCGAAGGGGGTCGGAACGTGGACTCCA	35
5[476]	5[475]	AATCAGATATAGAGAATAGTAAAAGTTACAAAATCGCAAGCA	42
5[497]	0[490]	CCGATAGGGTGAATAGAAAAGATAGCTGTTTCCTG	35
5[917]	0[910]	CGAACTGCCCGGAAATCGGTGGTTTTCCCAGTCAC	35
6[930]	5[940]	AGTATAGACCAACTTTGAAAGAGG	24
7[28]	4[42]	CGCAATAATAACGGAATACCCTAAGAAAAGAAAAAGTTTGAA	42
7[63]	2[56]	GATTAAGCTATCTTATATGCGTCTTCTGACCTAAATTAGGAGACCTCAA	49
7[483]	0[469]	TTATTAGACAAGGCTTATCCGTTGCCTGTTGTTATCCGCTCA	42
7[504]	2[497]	TCATAATCTTTCGATTGCGCCAGAAGTTTTGCCAGGCGTCCAATGTCAA	49
7[917]	7[940]	AGGATTAGGATTAGCGGGGTTTTG	24
0[90]	5[97]	CAGTTTGCGGAACCTGAAAAAAGCCGTCAATAGATTTTTTCATAAAGCC	49
0[132]	5[139]	ATAAATCAAGCCGGAACACCGACTTTACAAACAATGCTGATGTATTTAA	49
0[174]	5[181]	AAATCCTGGAGCGGACCACCAGAACGTTATTAATTTTAACCTGAGCCAG	49
0[216]	5[223]	AGTTGCACGCGTAAAACTGATGCGGAACAAAGAAAATAGTGAGTAATTC	49
0[258]	5[265]	ACGGGCAGGCGCGTCACAGACATTCCTGATTATCAAAAACATAATGCAG	49
0[300]	5[307]	TGCGTATTTCCTCGAGAGATAGATTGTTTGGATTATGTAAATACAAGAA	49
0[342]	5[349]	AGCTGCATAAAGGGACCAGTCCTACCATATCAAAAACAGTACAACCAAT	49
0[384]	5[391]	TGCGCTCAGTGTTTACGCTCAAATTGCGTAGATTTTTACATTCGGGTAT	49
0[426]	5[433]	CCTGGGGTCCATCACATTGCATAACAGTACCTTTTCAAAAGACCGTTTT	49
0[510]	5[517]	CATGGTCCCCCAAAAACTAGCATACTGCGGAATCGTTGCAAAGACAATG	49
0[552]	5[559]	TAAAGACAAACGTTGAGTCTGATGCTTTAAACAGTCGTTTACGGTCGCT	49
0[594]	5[601]	TGAGTAAAATCAGCTATTTTTAAATCAGGTCTTTAAGGAATTATCGTCA	49
0[636]	5[643]	CATAAATATTCGCGTTCAACCGGATTGCATCAAAATAGGAATAGCAACG	49
0[678]	5[685]	TGGGCACAATGTGAGTGAGAAAATATCGCGTTTTAGAACAACGAGGAAG	49
0[720]	5[727]	ATACCGAAACGGCGAAATGCAAAGCAAACTCCAACGACGTTGCGAAGGC	49
0[762]	5[769]	CCGCTTCATGGGCGTTCAACGGCTCCTTTTGATAATACCTTAAAAACAC	49
0[804]	5[811]	GCTGAATACGACAGAACATTATTAATTGCTGAATATGGGCTTAACAAAG	49
0[846]	5[853]	AAGTGGTTCCGGCACAATAAAATGCAACTAAAGTATAAGGCTTGTCGAA	49
0[888]	5[895]	GTGCCAATCGCCATCAATAAACAGTTGATTCCCAATCATTACAGGCGCA	49
2[97]	7[104]	CAGCAAACTAAAGGAGAGCAATAGCAAACGTAGAA	35
2[139]	7[146]	CAGTATTCGAACGTTCAGAGAAAAGAAACGCAAAG	35
2[181]	7[188]	CGAACGAGCGCTAGTGAACACAATCAATAGAAAAT	35
2[223]	7[230]	ATGCGCGCCACCACATAACATAAGGGCGACATTCA	35
2[265]	7[272]	TACGTGGACTATGGTGTTTAAGACGGAAATTATTC	35
2[307]	7[314]	GGCCAACTTAGAATCAGCCATCTTGAGCCATTTGG	35
2[349]	7[356]	CAGATTCATTTTAGCGAGCGTCACCATTACCATTA	35
2[391]	7[398]	CATTTTGTTATAATTGCACCCCATCGATAGCAGCA	35
2[433]	7[440]	CGCCAGCCGCAAATGCGGGAGTGCCTTTAGCGTCA	35
2[517]	7[524]	ATCGTAAAACAGGACAGCTTGCAAAATCACCGGAA	35
2[559]	7[566]	TGCCTGAAATATTTAAAAAAACAGAGCCGCCACCC	35
2[601]	7[608]	GGGTAGCTCATTTTAAGGAATCCTCAGAGCCGCCA	35
2[643]	7[650]	TATGATATCTGGCCTTTCAACCATTGACAGGAGGT	35
2[685]	7[692]	CAAAAGGGCGAGTACGTTAGTATTCACAAACAAAT	35
2[727]	7[734]	ATATTTTGATTGACCATAGTTCGCAGTCTCTGAAT	35
2[769]	7[776]	CCTTTATCATCGTACAGTACACTTTTGATGATACA	35
2[811]	7[818]	TACCAAATATCGGCGCCCAATGGTCAGTGCCTTGA	35
2[853]	7[860]	AATTAAGCCGCTTCCGCCACCCCCCCTGCCTATTT	35
2[895]	7[902]	TAACATCTCAGGCTGAGGTTTTATTAAGAGGCTGA	35
5[98]	0[91]	AACGCTCAATAATAGAGCCCCGTTGAGTGTTGTTC	35
5[140]	0[133]	CAACGCCGCTAATAGGCGAGACGGCAAAATCCCTT	35
5[182]	0[175]	TAATAAGAATTAACGGCGCTGGCCCCAGCAGGCGA	35
5[224]	0[217]	TGTCCAGAGAGAGAACCCGCCCCTGGCCCTGAGAG	35
5[266]	0[259]	AACGCGCGATTTTTTTGCTTTTTTTCACCAGTGAG	35
5[308]	0[301]	AAATAATAAATAAACAGAGCGGGGGAGAGGCGGTT	35
5[350]	0[343]	CAATAATACGCTAAACAGGAAAAACCTGTCGTGCC	35
5[392]	0[385]	TAAACCAGCTATTTCAGTGAGCACATTAATTGCGT	35
5[434]	0[427]	TATTTTCCCGACTTTAACCGTCATAAAGTGTAAAG	35
5[518]	0[511]	ACAACAAGGTTTATAGATTGTCTCGAATTCGTAAT	35
5[560]	0[553]	GAGGCTTATCTCCATGTTAAAGCTTGTTACCTCGA	35
5[602]	0[595]	CCCTCAGACAACTATTAACCACCTGGTTGGTGTAA	35
5[644]	0[637]	GCTACAGAAACAACTTCCTGTGCTGACGCATTTCA	35
5[686]	0[679]	TTTCCATTTCCAGAACAACCCAAGCAACTCGTCGG	35
5[728]	0[721]	ACCAACCCAGCCCTCGTAATGGGAGTGACTCTATG	35
5[770]	0[763]	TCATCTTTCGTCACACCGTGCCCGCCAAAATAACC	35
5[812]	0[805]	TACAACGATAGCAACTCAGGATAAGTGTCCTTAGT	35
5[854]	0[847]	ATCCGCGTCAGAACTGGTGCCGTGGATGTTCTTCT	35
5[896]	0[889]	GACGGTCTACTCAGGCGCAACTAAAACGACGGCCA	35
7[105]	2[98]	AATACATTAAGCCCAACAGTAGAACGCGAGAAAACAATACATGAGCCAG	49
7[147]	2[140]	ACACCACATTGAGCAACATGTAACTATATGTAAATTCGACAAAGGCGGT	49
7[189]	2[182]	TCATATGACGGGAGAGAATATGAGAGACTACCTTTTTAAAAGAAAATAC	49
7[231]	2[224]	ACCGATTCCTTTACACGACGAGCTGAGAAGAGTCACCACCAGGTCTTTA	49
7[273]	2[266]	ATTAAAGAAGAAACCTGTTTACCTTAGAATCCTTGGATGATGGTAAGAA	49
7[315]	2[308]	GAATTAGAGTTACAATCCCATAATAACCTTGCTTCTACTTCTACATTCT	49
7[357]	2[350]	GCAAGGCCTTACCACGGCTGTCTTTTTTAATGGAATTATTTGACATTGG	49
7[399]	2[392]	CCGTAATATTAGTTAGTACCGGAAAACAAAATTAATCAGGTTATACCTA	49
7[441]	2[434]	GACTGTAGAACCTCATCGTAGTTCAATTACCTGAGACATCGGATATTAC	49
7[525]	2[518]	CCAGAGCTTGTATCCCATCGCATAGCGAGAGGCTTTCATAAAAACGGTA	49
7[567]	2[560]	TCAGAACGTTGAAAGCAGGGAACTATCATAACCCTTCAGAAAAGGTCAT	49
7[609]	2[602]	CCAGAACGAAAGGACAGCGAATACATAACGCCAAACCCTGACCCGGAGA	49
7[651]	2[644]	TGAGGCATTTTGCTAGGCTTTCATCAGTTGAGATTAGATTAACCATCAA	49
7[693]	2[686]	AAATCCTGTCGTCTTAAACGGAAAACGAACTAACGATTCGAGAAAGATT	49
7[735]	2[728]	TTACCGTCCACAGATAAAACGATTATACCAGTCAGAGGTCAGCCCTCAT	49
7[777]	2[770]	GGAGTGTCTGAGTTTGACCCCTAATCATTGTGAATGAGGTCAGGAGAAG	49
7[819]	2[812]	GTAACAGTTCAGGGGAGATTTAACGAGTAGTAAATTAATGCTTCGGTTG	49
7[861]	2[854]	CGGAACCGCCACCCACCTGCTGCTCATTCAGTGAACGGTGTCTTAGCAA	49
7[903]	2[896]	GACTCCTATCACCGAATCATACAAGAACCGGATATTTCTGCGGTAGCAT	49
0[69]	5[76]	TATTAAAAGGTGCCTTGCTGACACTAACAACTAATAATTTCATTATACA	49
0[111]	5[118]	AGATAGGCGATTTAACGCTGATTGAGGATTTAGAAAGACAAAGGGCTTA	49
0[153]	5[160]	CCGAAATAAGGAAGAGAGGTGCTCGTATTAAATCCGTTATATAATTTAG	49
0[195]	5[202]	CTGGTTTGCAAGTGGCCATTATTTGAGTAACATTATAGGTCTAAAGTAC	49
0[237]	5[244]	TTCACCGGCGCTTAGCTATTAAAGGAGCGGAATTATTAAGACCAATAAA	49
0[279]	5[286]	GTTTTTCGACGAGCTGAAAGCGCAATTCATCAATAAATTTTCTCAACAA	49
0[321]	5[328]	AACGCGCGGAGCTAAAAAGGGGAATAATGGAAGGGGTGAGTGCCTAATT	49
0[363]	5[370]	AGTCGGGCGGTACGATTATTTCACGTAAAACAGAAGAATTACCTTTCCT	49
0[405]	5[412]	GCTAACTGCCACCGCATGGAATAACGTCAGATGAAACATCAACACTCAT	49
0[447]	5[454]	CCGGAAGTGTAGCACAGAACAGAGAAACAATAACGTATTCATGAATCAT	49
0[531]	5[538]	TACCGAGATAAGCAATCGATGTATTCATTGAATCCAACCAAACCACGCA	49
0[573]	5[580]	ACGTGGTATTCGCAGGCTATCACGAGAATGACCATGAGCAACGTTAAAG	49
0[615]	5[622]	CTGACCTATAGGAAATTAATGTATTATAGTCAGAAATGCAGAAGACAGC	49
0[657]	5[664]	TGAATCGAGCCAGCCAAATCAGAGGAAGCCCGAAAAAAGATTGAGGACT	49
0[699]	5[706]	CATCTGTGTCGGATTGTAGGTCTTCAAAGCGAACCCGTTAATGTAAAAT	49
0[741]	5[748]	TCGCCCTGGATAGGTTTAGAAGATTAGAGAGTACCCTGGCTCAAAGAGG	49
0[783]	5[790]	CAATGTCATCTGCCTTTTGCGTTTTTGCGGATGGCTCAACTTCAGCGAT	49
0[825]	5[832]	CACGACTAGATCGCAGCTAAAGTAGCTCAACATGTACACCAGGTATCAT	49
0[867]	5[874]	AGCCAGGGGAAACCCAAAGAATGGAAGTTTCATTCCAAAGCTCCATGTT	49
2[76]	7[83]	CATCACCGTAAAGCGCAATAGACTCCTTATTACGC	35
2[118]	7[125]	CAGTGCCGAGCTTGATTGAGTACATAAAGGTGGCA	35
2[160]	7[167]	ATAAAACGGAAGAAGAGGGTAGGAATAAGTTTATT	35
2[202]	7[209]	AAACATCTAGCGGTGCATTAGGTTTACCAGCGCCA	35
2[244]	7[251]	TTGAATGATGCGCCATAGCAGGAGGGAGGGAAGGT	35
2[286]	7[293]	TCTGACCACGTATATCCAAATGTGAATTATCACCG	35
2[328]	7[335]	CAGTAATAACAGGAATTTGCCAGCCAGCAAAATCA	35
2[370]	7[377]	GAAATGGCCAGAATCCTGAATCGGAAACGTCACCA	35
2[412]	7[419]	AAACGCTAGTAAAAAATCAAGCAGTAGCGACAGAA	35
2[454]	7[461]	TAATATCATACTTCTTTTAGCGCGCGTTTTCATCG	35
2[538]	7[545]	CAAGAGAAATATTTCCTTTAACACCACCGGAACCG	35
2[580]	7[587]	CTACAAATTAAATTTTTTCACCGCCACCCTCAGAG	35
2[622]	7[629]	CTGATAACGCCATCGAGAATACACCACCAGAGCCG	35
2[664]	7[671]	AGACAGTTTTCATCTATGGGAGGTCAGACGATTGG	35
2[706]	7[713]	AGTAATGTCTCCGTAAGTTTTCATTAAAGCCAGAA	35
2[748]	7[755]	AAAAATTTCACGTTTAGCATTTCCAGTAAGCGTCA	35
2[790]	7[797]	GTAATACAGTTTGACGTAACAACTGGTAATAAGTT	35
2[832]	7[839]	AGCATAAACTCCAGCCTCATTTGCCCGTATAAACA	35
2[874]	7[881]	GGCAAGGAGGCAAACAGAACCTATTATTCTGAAAC	35
5[77]	0[70]	AATTCTTTGAAATAACTAAATGAACAAGAGTCCAC	35
5[119]	0[112]	ATTGAGACACAAGAACGGGGAAAAAGAATAGCCCG	35
5[161]	0[154]	GCAGAGGAAAGTCAAGCGAAAGTTTGATGGTGGTT	35
5[203]	0[196]	CGACAAAGGGAAGCCACGCTGGCAAGCGGTCCACG	35
5[245]	0[238]	CAACATGAATGAAAGCTACAGACAGCTGATTGCCC	35
5[287]	0[280]	TAGATAAATCCCAAACGTGCTTGGGCGCCAGGGTG	35
5[329]	0[322]	TACGAGCGAGCCTAGGCCGATTTAATGAATCGGCC	35
5[371]	0[364]	TATCATTATTTTATCCTGAGAACTGCCCGCTTTCC	35
5[413]	0[406]	CGAGAACAGCCTTAGAGTCTGTGCCTAATGAGTGA	35
5[455]	0[448]	TACCGCGCGAGGCGTTTGATTACACAACATACGAG	35
5[539]	0[532]	TAACCGAAAAGGAGAAATTGTGGAGGATCCCCGGG	35
5[581]	0[574]	GCCGCTTAATAATTTTTGTTAACAGGGCTTAAGCT	35
5[623]	0[616]	ATCGGAAGCGGAGTAAAAATACATTTCTCCGAACT	35
5[665]	0[658]	AAAGACTTTTTCTGAACATTAGAATATAGGGGCCT	35
5[707]	0[700]	ACGTAATCGATCTAGGGAACACAGTGCGGCCCTGC	35
5[749]	0[742]	CAAAAGAACGCCTGGGTGTAGTAATCTATTTACGC	35
5[791]	0[784]	TATACCACATGTACGGGGACGTGTCAACCTTATGA	35
5[833]	0[826]	CGCCTGACCACCACCCAGCTTTGTGAATTCATGCG	35
5[875]	0[868]	ACTTAGCCCACCCTGCGCCATGCTTTCTCAGGAGA	35
7[84]	2[77]	AGTATGTGAAACAAACCAGTAAATATATTTTAGTTAGATTAGTCTAAAG	49
7[126]	2[119]	ACATATAGATAACCATCGCCACAAATCCAATCGCAGTATTAGCCTGCAA	49
7[168]	2[161]	TTGTCACCCTGAACCATTTTCCCGGCTTAGGTTGGTTTGCCCGCAGAAG	49
7[210]	2[203]	AAGACAAAAAAACAAGGTAAAATTTATCAAAATCATCATTTTAGCCCTA	49
7[252]	2[245]	AAATATTCGTCAAATTCAGCTAGCGATAGCTTAGATCATCATAATATTT	49
7[294]	2[287]	TCACCGAATTATTTGTCCTGACGTCGCTATTAATTTAATCCTGAACCCT	49
7[336]	2[329]	CCAGTAGCTTTCCAATGTAGAATAAATCAATATATTTAGAACACACGAC	49
7[378]	2[371]	ATGAAACAGCTACACCAAGAATAACAATTTCATTTATAAAGAATCGTCT	49
7[420]	2[413]	TCAAGTTGTTTTGAAAGCAAGAGATGATGAAACAATATACAGACAGGAA	49
7[462]	2[455]	GCATTTTAAGAACGCCCAATAGCGCAGAGGCGAATGATTCGCTTGCTGG	49
7[546]	2[539]	CCTCCCTGGCTCCATATATTCCAGACGACGATAAACCCTCAAGAGCAAA	49
7[588]	2[581]	CCACCACTGCGAATTTGCGGGACGAGGCATAGTAAAAATCAAGAGAGAT	49
7[630]	2[623]	CCGCCAGAGTTTCACGAGGGTACCACATTCAACTAGCAAAGCGTTCTAG	49
7[672]	2[665]	CCTTGATAAATGAATTTTCATATTATTACAGGTAGGACTTCAAGGCCGG	49
7[714]	2[707]	TGGAAAGAGCGTAAGCCACTAGGAAGAAAAATCTAAGACCGGATGCCTG	49
7[756]	2[749]	TACATGGAACTACAATACACTTGCGATTTTAAGAATTTAATTCAAGGAT	49
7[798]	2[791]	TTAACGGAGGAACCAGCGCGAGAGATGGTTTAATTTTAGAGCTGACCCT	49
7[840]	2[833]	GTTAATGCTCAGAGTAAATTGTGCCCTGACGAGAATTTAAATGCCTCAG	49
7[882]	2[875]	ATGAAAGAGTACCGCGGAACGCCAAATCAACGTAACATATAATCATACA	49

biotin-modified
CCGTGTGATAAATAAGGCGTTAATTTTTTT-Bio
AGATAGCCGAACAAAGTTACCAGTTTTTTT-Bio
Bio-TTTTTTTTTTTATGGCCCACTACGTGAACC
Bio-TTTTTTTTTTTAACAGTTGAAAGGAATTGA

digoxigenin-modified
CAGCTGGCGAAAGGGGTTTTTTT-Dig
CGCAAATGGTCAATAACCTGTTTTTTTTTT-Dig
Dig-TTTTTTTTTTTCAGACCAGGCGCATAGGCTGGCTGAC
Dig-TTTTTTTTTTTTTAAGTGCCGTCGAGAGGGTTGATATA

10 helix bundle

Start	End	Sequence	Length
1[31]	8[38]	CCACTACGTGAACCATCACCCAAATGCGATTATACCAAGCGCG	43
1[385]	8[399]	AGAAGTGCGGTTTAGTCAGGACGCACCACCAGAGCGCCTTTA	42
0[55]	6[38]	AAAGAACCGCCCTGACCATAAATCAAAAATCA	32
0[69]	9[76]	AAGAGTCTTTGGGGTCGAGGTAACACTCCTCCATG	35
0[90]	9[97]	AGTGTTGAGCACTAAATCGGAAGAGGCACGAGGCG	35
0[111]	9[118]	GAATAGCAGGGAGCCCCCGATAAGGCACTAAGGGA	35
0[132]	9[139]	AAAATCCTTGACGGGGAAAGCAAAATACCTTTGAA	35
0[153]	9[160]	GATGGTGCGTGGCGAGAAAGGGGAAGTTCGGTGTA	35
0[174]	9[181]	CAGCAGGGAAAGCGAAAGGAGGGACTAAAGGCTGG	35
0[195]	9[202]	GCGGTCCTAGGGCGCTGGCAACAACGGCGAGTAAT	35
0[216]	9[223]	GCCCTGAGGTCACGCTGCGCGACAGCATGATATTC	35
0[237]	9[244]	CTGATTGCACACCCGCCGCGCCGTCACCCGTAACA	35
0[258]	9[265]	CACCAGTGCCGCTACAGGGCGTAAAGGCGTGAATA	35
0[265]	7[265]	TTCTTTTACCGAGCTACGAGGACTAAAG	28
0[279]	9[286]	CGCCAGGTGGTTGCTTTGACGTCGCTGAGAGAAAC	35
0[300]	9[307]	AGAGGCGATAACGTGCTTTCCACGCATATAAATTG	35
0[321]	9[328]	TGAATCGAATCAGAGCGGGAGCAATGACTAATTTC	35
0[342]	9[349]	CTGTCGTGGAGGCCGATTAAATGATACCTGAATTA	35
0[363]	9[370]	CCCGCTTTAGACAGGAACGGTAGGTGAAAAGAACT	35
0[405]	9[412]	TTGAGTAAATCAGTGAGGCCAAAAAGGACGCCGCC	35
0[426]	9[433]	AACGTTAAAAGAGTCTGTCCAAATCTCCGTTGAGG	35
0[447]	9[454]	TCGTATTAATTAACCGTTGTATAATAATGGCCTTG	35
0[468]	9[475]	CTTTACATTCTTTGATTAGTAAACAACTATAAATC	35
0[489]	9[496]	TGAGGATCACTTGCCTGAGTAAGCGGAGAATGGAA	35
0[510]	9[517]	GCCGTCATCAAACTATCGGCCTAAACAAATTTACC	35
0[531]	9[538]	ACTAACATAATATCCAGAACAATTTTCTCATACAT	35
0[552]	9[559]	ATCTAAACGCCAGCCATTGCATTTCCAGCAGGAGT	35
0[573]	9[580]	TGAAAGGAAACGCTCATGGAAACGATCTTTTTAAC	35
0[594]	9[601]	GTCAGTTCATTTTGACGCTCAACAGCCCGAGTAAC	35
0[615]	9[622]	CAAACCCGAAATGGATTATTTAACGCCTCAGTTAA	35
0[636]	9[643]	ACCTTGCCAGATTCACCAGTCTTCGTCATTCGGAA	35
0[657]	9[664]	AAATGAACAGTAATAAAAGGGCCATGTAACATGAA	35
0[678]	9[686]	GCCACGCGGCCAACAGAGATAGATAGCAGAGACTCC	36
0[699]	9[706]	ATTAACATCTGACCTGAAAGCGCCACCATAGGATT	35
0[720]	9[727]	AACAGAGTACGTGGCACAGACCTCAGAACAGTACC	35
0[741]	1[756]	CGAACCATTGAATGGCTATTAGTCTTTAA	29
0[748]	7[748]	TACCGAAAAACAAACGGAAATTGGTTTA	28
3[31]	0[37]	TTCTAATCTATTTACGCTGTGGACTCCAAC	30
2[55]	4[34]	GTCCTTAAGAGAATGAGGGTAGCTATTTTTGAGAGA	36
2[391]	7[391]	ATTGCATCTGCCAGGAGTGAGCTAACTTGGGAAGATCATTTG	42
2[748]	4[742]	AACAGTAACGGGTACGTAGGA	21
5[24]	2[42]	ATTGCCTGAGAGTCTGGAGCAAACAGTGCTGA	32
5[63]	3[69]	CGGTAATCGCACGATCTATGA	21
5[525]	3[531]	GCATTTTGCAAATCATATCAA	21
4[69]	8[63]	AAATTAAAAGAGGAAGAAAACCGACCTGATCTTTG	35
4[90]	8[84]	TATTCAACAAATATCTCAAATGCCGGAAAAAGAAT	35
4[111]	8[105]	GTCAAATAGCTTCAATAAATATCAATCACAACCTA	35
4[132]	8[126]	GGGTGAGGGAAGCAGTCCAATTGACCAAGTAATGC	35
4[153]	8[147]	TGTGTAGAGGATTAAAAATGTAGATGAATCCATTA	35
4[174]	8[168]	TTAAATGTTGCTCCTTTGCCAGGCGCATAGACTTT	35
4[195]	8[189]	TTTTTAGCATTTTTAGAGGCTTCATCAATACAGAG	35
4[216]	8[210]	ATTTCAAGCTTAATACGATAAAGAACCGCGGAACG	35
4[237]	8[231]	ACTTTTGCTGTAGCATAACCCAAATCAACTCAGCA	35
4[258]	8[252]	AAAACATATATGCACATAGTATCATTCACGCTTTT	35
4[279]	8[273]	AAAGCTATCTGGAAACGCCAACCCTGACGGCTTGC	35
4[300]	8[294]	AGCAATAAACAGTTTTCAACTCGAGTAGACCGATA	35
4[321]	8[315]	GGCAAAGCGAACGATTGAGATGATGGTTAACAACC	35
4[342]	8[336]	TCCAATAACCATTAACAGGTAATCATTGGATAGTT	35
4[363]	8[357]	CTAATAGATGGTCAAACTAACCGATTTTTTTCTTA	35
4[384]	8[378]	GAAAAGGTATATTTAAAATCTTATACCATCAGCTT	35
4[405]	3[405]	GAATTAACTGAAGCTAATTACTAGAAAAGAATAAAACAAAGA	42
4[426]	8[420]	AGGGAAGTGAGCGCCAGAGCCACAGGAGAAAAAAA	35
4[447]	8[441]	CAGAGAGTAACCCACACCCTCGACGATTTTTTTCA	35
4[468]	8[462]	AAATGAAAGCCCAAAACCGCCCAAACAAAAAGGAA	35
4[489]	8[483]	CGATTTTAACAATGCGGAACCAAGCCAGTGAGAAT	35
4[510]	8[504]	TATCCCAATCTTACTCTTTTCTCTCTGACTTTCAA	35
4[531]	8[525]	AAAATAAAGAAAAGCCCCCTTTAAGCGTGTATGGG	35
4[552]	8[546]	AGAGCCTAACAAAGCATCGGCGATGATAACGTTAG	35
4[573]	8[567]	AACGCTACCGAGGACGTCAGATAATAAGAAAGTTT	35
4[594]	8[588]	AATTTTAGGAATACCAGAATCGTGCCTTTCATAGT	35
4[615]	8[609]	TGCTATTATGATTACAGCACCGTATAAAGTAGCAT	35
4[636]	8[630]	AAGCCTTGCAGTATCACCAATTGCCTATCCAGTAC	35
4[657]	8[651]	CCCGACTAAAATACCATTAGCTTCTGAACCGTAAC	35
4[678]	8[672]	GCGAGGCCAACATAAATCACCAGAGGCTAGCCCAA	35
4[699]	8[693]	CTTATCCAGACACCTTTGGGAGAAGGATCCCTCAT	35
4[720]	8[714]	AAGCAAATTTTGTCCACCGTCTTTTGCTCCGCCAC	35
4[741]	8[735]	ATCATTAATTCATATATTCATTAAGTGCTCAGAAC	35
4[769]	5[759]	GCAAGCCGTTTTTATTTTCATTTAAACCAAGT	32
7[44]	5[62]	GGATTGCATCAAAAAGATTTGCCGGACGATGAA	33
7[77]	3[90]	AAGACTTCCGTTCTCTAGCATGTCAATCCTAAGTGCCCTGCC	42
7[98]	3[111]	TAATTCGCACCATCCCCCGGTTGATAATGAAGCCACGTCGGT	42
7[119]	3[132]	CCAGACCAAAGGCCGCCCCAAAAACAGGCAGTGCCGGGCCTT	42
7[140]	3[153]	ACAGGTCGTAAAGATATAAGCAAATATTACGACGTATTTCAC	42
7[161]	3[174]	CCTTTAACAATGCCTAAACGTTAATATTAACGCCACGAACTC	42
7[182]	3[195]	AAGAGGTAACCCTCAATTCGCATTAAATTGCAAGGGTGTAAT	42
7[203]	3[216]	GCTTAGACGCAAGGAAATCAGCTCATTTGCTGGCGTAAGCTA	42
7[224]	3[237]	TATAATGCGGGAGAAATAGGAACGCCATGGCCTCTCCTCGAT	42
7[245]	3[258]	GTTTTAATATGACCAATTCGCGTCTGGCTTGGGAACCCGGGT	42
7[266]	3[279]	TACGGTGAATCGGTTAGCCAGCTTTCATGCCATTCCGTAATC	42
7[287]	3[300]	TCCATATAAGCCTCAAATGTGAGCGAGTAAACCAGTTCCTGT	42
7[308]	3[321]	AATTCTGAATTAGCCGTCGGATTCTCCGCGGCACCCGCTCAC	42
7[329]	3[342]	TAGTTTGAATCATAAAACGGCGGATTGAATCGCACTACGAGC	42
7[350]	3[363]	TTCGCAATAGTAGCGGGATAGGTCACGTGACAGTAGTAAAGC	42
7[371]	4[385]	GTTTAGCTGGCATCGATGGGCGCATCGTAACCGCAGCGAGCT	42
7[392]	2[392]	GGCACCCTGAACAACGCCACCAGAACTTTTGCGGACACCGGA	42
7[413]	3[426]	GGGTAATCGCATTATTAGTATCATATGCGATAAATGCGGAAT	42
7[434]	3[447]	AGAGAGAAATAACAAAATTCTTACCAGTGGTTTGATGATTAT	42
7[455]	3[468]	TGAGTTAAATAGCACAACGCTCAACAGTATCTTCTTCATCAA	42
7[476]	3[489]	AGCAAGATTGTTTAAATTGAGAATCGCCAAATATATTTGGAT	42
7[497]	3[510]	AATAGCTATCCAAAACAACGCCAACATGAGAACGCATGGAAG	42
7[518]	5[524]	CTTTTTAACAGCCAGGCAGAG	21
7[539]	3[552]	ATAGCCGAATTTGCGTAATAAGAGAATATAACTATAAAACAG	42
7[560]	3[573]	AAGGAAAACGAGCGCCGACAAAAGGTAATCCGGCTCGTAGAT	42
7[581]	3[594]	TAATAACTCCTGAACTGTCCAGACGACGTGAGAGATCAGATG	42
7[602]	3[615]	AACTGGCTTGCACCACAACATGTTCAGCAATTTATGTACCTT	42
7[623]	3[636]	TTATTACAAATCAAGAACGCGCCTGTTTCGCTGAGACAATAA	42
7[644]	3[657]	AACGTAGTGCGGGAATAGATAAGTCCTGTAGCGATTGCTTTG	42
7[665]	3[678]	AAGGTGGGTTTTAGAAAATAATATCCCACCCTTAGAAATCGC	42
7[686]	3[699]	AACGCAAGGTATTCTTACGAGCATGTAGTCGTCGCTTCATTT	42
7[707]	3[720]	AAGTTTATCAGATATCAATAATCGGCTGGAATAACAAAGAAG	42
7[728]	3[741]	ATAGAAACCGCGCCTTATCATTCCAAGACATAAATATCAAGA	42
6[763]	3[756]	GAAGGTAAATATTGAATTAATTA	23
9[44]	2[56]	TAAATTGTGTCGAAATCCGGAGAATGGAGTGACCTTAAGT	40
9[77]	4[70]	TTACTTAGCTTTAAAGTGCGGGTTGTGAATTCATGCGTAAAAAGCTGAT	49
9[98]	4[91]	CAGACGGTTCATTGAGCAACTGGGTGGATGTTCTTATATGTAAATATGA	49
9[119]	4[112]	ACCGAACACTGCGGAATATAGAAGCTTTCTCAGGACAGAAAAGGAGACA	49
9[140]	4[133]	AGAGGACTTAGACTCTGACGCTGTAAAACGACGGCAAGATTGTTCAAAA	49
9[161]	4[154]	CAGACCAGAGGGGGATTTCTCGGGTTTTCCCAGTCTAAATTGTGAGTAA	49
9[182]	4[175]	CTGACCTTTTGCAACTGGTTGCGATTAAGTTGGGTTTGTTAAATATATT	49
9[203]	4[196]	CTTGACAAAACCAACAGGGCTAAAGGGGGATGTGCTTTTGTTATAAAAA	49
9[224]	4[217]	ATTACCCTCGTTTACTTGTTATCGCTATTACGCCATTTAACCAGCCTTT	49
9[245]	4[238]	AAGCTGCAGAGCAAGAGGATCGGGCGATCGGTGCGCAAAAATCTGTAAT	49
9[266]	4[259]	AGGCTTGAAGGAATTCGAATTAGGCTGCGCAACTGCTTCCTGTGTACCA	49
9[287]	4[280]	ACCAGAAAATGCAGTAGCTGTGCAAAGCGCCATTCCAACATTAGAGCAT	49
9[308]	4[301]	GGCTTGATTAGGAATGTTATCGCTTCTGGTGCCGGAACAACCAAAATTA	49
9[329]	4[322]	AACTTTAGAAAGATCACAACATCCAGCCAGCTTTCTGGGAACCAGGCAA	49
9[350]	4[343]	CCTTATGGGAACAAATAAAGTTCGGCCTCAGGAAGCCGTAATATTAACA	49
9[371]	4[364]	GGCTCATACGTTAAGCCTAATTTTGAGGGGACGACTGGTGTAAATTCTA	49
9[413]	4[406]	AGCATTGACCACCCAGAAGGAAAGGCGTTAAATAAAGCCTGTGACGGGA	49
9[434]	4[427]	CAGGTCAAGAACCGATATTCCAATACCGACCGTGTGTTATACTAAAAAC	49
9[455]	4[448]	ATATTCATCCCTCATGGCAATGACCTAAATTTAATATAAAGCGCCTTTA	49
9[476]	4[469]	CTCATTAAGAGCCACTGATTGTTTTAGTTAATTTCAGGGCTTACGTCAA	49
9[497]	4[490]	AGCGCAGATAATCACTGAATAGAGAAAACTTTTTCATATTTATAAGAAA	49
9[518]	4[511]	GTTCCAGATTAGCGACCTACCCAATCGCAAGACAATAATTTATATTATT	49
9[539]	4[532]	GGCTTTTATTTTCGTGCACGTATGTAAATGCTGATCGAGCCACAGTTAC	49
9[560]	4[553]	GTACTGGCTGTAGCGAAATTGTAGGTTGGGTTATATAAAGTATCTTTCC	49
9[581]	4[574]	GGGGTCAAAGTTTGTTTAACGCTACCTTTTTAACCAGTAATTTCTTACC	49
9[602]	4[595]	AGTGCCCGTAATCAAGTAACACAAAATCATAGGTCACAATAACAGCTAC	49
9[623]	4[616]	TGCCCCCGAAACCAGGGAGAAAAGAGTCAATAGTGTAATGCAGATTAGT	49
9[644]	4[637]	CCTATTAAAGGCCGGCCTGATAGCTTAGATTAAGAATCAACAGGTTTTG	49
9[665]	4[658]	AGTATTAAGTAGCAAGTTACAAATCCTTGAAAACAAACAAGACGAACCT	49
9[687]	4[679]	TCAAGAATTAGAGCGAATTATATTAATTAATTTTTCCTAATTAAGAAC	48
9[707]	4[700]	AGCGGGGACCGACTCTGAGCACTTGCTTCTGTAAAAAACCAATAGAAGG	49
9[728]	4[721]	AGGCGGATAAAGGTAACAAACCAATATATGTGAGTTCTTTCCCAATAGC	49
8[62]	0[56]	ACCCCCACAAGTTTCACTATT	21
8[83]	7[76]	ACACTAAGCCGTAATTCCAGTTTGGAACTACCGACACAGTTCAGCCCGA	49
8[104]	7[97]	AAACGAAACCCTAACCGAGATAGGGTTGATCTGTAAATCCCCCGCGTTT	49
8[125]	7[118]	CACTACGTTAGAGCCTTATAAATCAAAAGGGCACGAATCGTCAAGCGAA	49
8[146]	7[139]	AACGGGTCGGCGAAGTTCCGAAATCGGCGAATCGGGGATAGCAACTCCA	49
8[167]	7[160]	TTCATGAAAGGGAACGAAAATCCTGTTTATAAATCTAATAGTGAGAGTA	49
8[188]	7[181]	GCTTTGACGGGCGCACGCTGGTTTGCCCTGACCTCAAGAAGTTTTTGAT	49
8[209]	7[202]	AGGGTAGGTGTAGCGAGAGTTGCAGCAAGAGTAAAAATAGCGGCGGATG	49
8[230]	7[223]	GCGAAAGTAACCACCCCTTCACCGCCTGCGTGGTGCCAGACGTGCTGAA	49
8[251]	7[244]	GCGGGATTTAATGCGAGACGGGCAACAGAAAGACGCACTATCTCAACAT	49
8[272]	0[266]	AGGGAGTCGTACTAGTGGTTT	21
8[293]	7[286]	TATTCGGAGCACGTGTTTGCGTATTGGGATGGTCAATACATAGTTTCAT	49
8[314]	7[307]	ATCGCCCTCGTTAGGCCAACGCGCGGGGGTGAAATTACCACAGATTCCC	49
8[335]	7[328]	GCGCCGACTAAACAGCCAGCTGCATTAAAATTCCATCATCAGGTAGATT	49
8[356]	7[349]	AACAGCTGGGATTTTCCAGTCGGGAAACCGGAAGCCATTATTGATACAT	49
8[377]	7[370]	GCTTTCGACGCCAGCGTTGCGCTCACTGCTGGGGTTAAAACGATAACCT	49
8[398]	1[384]	ATTGTATTTTTTATACATTATCATCACATTAATTGAATCCTG	42
8[419]	7[412]	AGGCTCCCCGAGTATTAATTTTAAAAGTAACCACCTCAGAGCAGTCAGA	49
8[440]	7[433]	CGTTGAATCACGCAAAATCCTTTGCCCGTATCATCCCACCCTTAATATC	49
8[461]	7[454]	TTGCGAAGCAATACAACAATTCGACAACCAGATGAGAGCCGCCAAGAAT	49
8[482]	7[475]	AGAAAGGATAACATTTAGAAGTATTAGATATAATCCCACCGGTAATAAG	49
8[503]	7[496]	CAGTTTCGAAGAACATAGATAATACATTTATACTTAAATCACAAATAGC	49
8[524]	7[517]	ATTTTGCTTGCTGGACTAATAGATTAGAGGTTAGATTTGCCACGAAGCC	49
8[545]	7[538]	TAAATGAATATTACATATCTTTAGGAGCAATTATTGTCATAGTAAGCAG	49
8[566]	7[559]	TGTCGTCACAGGAAAATTGAGGAAGGTTAAATAAAGCGTTTTTTACCAG	49
8[587]	7[580]	TAGCGTAATACCTAGGCAAATCAACAGTTTTCAGGCCTTTAGAACGCAA	49
8[608]	7[601]	TCCACAGATCGTCTTCAATCAATATCTGAATATACGTAGCGACCAAAAG	49
8[629]	7[622]	AAACTACACATTGGTGAACCTCAAATATTTACATCTCGATAGAGACTCC	49
8[650]	7[643]	ACTGAGTACACGACAAATCTAAAGCATCCGGATTCGAAACGTGTTAGCA	49
8[671]	7[664]	TAGGAACACATTCTTGAGAGCCAGCAGCAATACCACCATTACATACATA	49
8[692]	7[685]	TTTCAGGGAACCCTCCGCCTGCAACAGTGCAGAGGCCAGCAATAAAAGA	49
8[713]	7[706]	CCTCAGAGTAAGAAGTGAGGCGGTCAGTCAATTACTGAGCCAACGGAAT	49
8[734]	7[727]	CGCCACCAATATTTCCAGCAGAAGATAAATGATGAGAATTATACAATCA	49
8[763]	9[748]	AGGAGGTTTAGTACCGCCACCCCGTCGAG	29

biotin-modified
GTCAAAGGGCGAAAAACCGTCTCCCTCC-Biotin
ATTGTCAACCTTATGACAATGTCCCGCCATCTC-Biotin
Biotin-ATTCTCATACCTAATATTATAGTCAGAAGCAAAGC
Biotin-ATATCTAATCCTCTGAGATTTGTATCATCGCCTGA

digoxigenin-modified
Dig-TATTACCCACCCTACCCTAAAACATCGCCATTAAAAA
Dig-TACCACAATTCATTTTTGAATTACCTTTTTTAATGGA
CCAGCGCCAAAGACAAAAGGGCGACATTCATCTTCTA-Dig
AGGGTTGATATAAGTATAGCCCGGAATAGGACTCCTT-Dig

12 helix bundle

Start	End	Sequence	Length
0[69]	5[69]	CGTCAAAGTGCCGTTATTTACCTGAAAGGATTGTTAGAAATT	42
0[321]	5[321]	GCGTATTAGCTAAAACGGTACCAGTTGGTATCTAAATATATG	42
0[342]	11[356]	ACGCGCGAACAAGGCGATTAAAGTAAATCACCAGAGCCGCCG	42
0[594]	5[594]	CGTGGTGGCCATCTAAACGGCGTTAAATCATCCAAGCGAACG	42
0[622]	5[615]	GTGTAATGAGTAAAGGTGGGCCGTCGGAACCAATATACTAATGACCATT	49
0[639]	1[650]	CTCTGACCTCCTTGAATCGGCTGACGCATTT	31
1[31]	11[62]	CCATCACCCAAATCAAGTTTTTTGGATTATTCCAGCAAAATCACCA	46
2[76]	11[83]	AATGGATAAAGCACGGAAGGTCATTACCATTAGCA	35
2[328]	11[335]	GACAGGACAGGAGGAGCCGAACAGAGCCGCCACCA	35
2[349]	9[349]	GGGGATGTGCTGGGAATCAACCGTTCTAAGGACAGAAACAAC	42
2[601]	11[608]	TGGGAACGTAAGCAATAAGTATATTCTGAAACATG	35
2[629]	11[622]	AGCGAGTAACAACCACGAATAAGTGCCGTAAGAGG	35
3[42]	8[39]	CAGAGATAGAACCCTATCCTGAATCTTACCA	31
4[629]	9[629]	GGTGGCATCAATTCGGAACGCAAAGACTGTCACCCTCAGCAG	42
5[42]	6[39]	TTGCACGTAAAACACGACCGTGTGATAAATA	31
5[70]	2[77]	GCGTAGAAAATTTATGCGTTATACAAATGTTGCTAATACGTGCGTCTGA	49
5[322]	9[328]	TGAGTGAATAACATGAATTGAGGAAGGTCAAATCATACGAGCAGTAAGC	49
5[343]	2[350]	AAGCCCGGGATTGCATAGGCTGGCTGACTTGAAAGGCTGATAGCGAAAG	49
5[595]	2[602]	AGTAGATTAAGAGCACTAACGGAACAACGAGGAAGTTTTTTATTCTCCG	49
5[616]	7[629]	AGATACAAAAAGGACAGGTAGAAAGATT	28
6[83]	9[76]	CTGACCTTTTTCAGATCAATATAATCCTCGTAAGATTTTGCAATCCCAA	49
6[328]	6[329]	TTCTGTACCATCCTAACAGACCAGGCGCATCAAAAAGCTTGC	42
6[356]	5[342]	GCAAAGCAAAGACTATCACCATCAATATGATATAGTAAGAGG	42
6[608]	9[601]	GGCATAGTTAGTTTAGTAGTAGCATTAACAGCTCATTTCCATAAAGGCC	49
6[636]	5[639]	AGATACATAACGCCTTTCGCAAATGGTCAAT	31
7[49]	2[49]	AAAAGCCTGTTTAGACAATTTTTCTGACATTGGCAGATTCAC	42
7[63]	4[56]	TATCATAATGGTTTGAAATACGAAATAATGGATTA	35
8[636]	3[639]	AGGCTTTGAGGACTCATCAAAAATAATTCGC	31
9[49]	0[42]	AAATAAACAGCCATGACGGAAGGTCGAGGGGCGAAAAACCGTCTATCAG	49
9[77]	0[70]	TCCAAATGAGGGAGTAAATCGAACGTGGACTCCAA	35
9[329]	2[329]	AGTGGGATTTTGCTATGAACGGTGTATTACAGTTGGATTTTA	42
9[350]	0[343]	TTTCAACAGACGTTGTTGGGTTAATGAATCGGCCA	35
9[602]	0[595]	GCTTTTGGGTTGATACTCGTCCAGGGCTTAAGCTA	35
11[39]	10[28]	GAATTAGAGCATTAAAGGTGAATTATCACCG	31
11[63]	8[63]	GTAGCACAAATATTATTATTTCCCAGCT	28
11[84]	6[84]	AGGCCGGACCGATTAAGAAACAAGATTATCTTACCTCATCTT	42
11[336]	0[322]	GAACCACGAATTTTCTGTAATCCGATTAGGGAGAGGCGGTTT	42
11[357]	6[357]	CCAGCATTCTTTCCAGTTTCAACCAACTCTTCATCGTCAGAA	42
11[609]	6[609]	AAAGTATTCGAGAGCGGGATCTTTTCATATTATTAATTACGA	42
11[623]	0[623]	CTGAGACTCCTCAAGTACCAGGCGGATATAGGGGCCTGGTTG	42
0[90]	5[90]	ATTAAAGGAACCCTGCTCAATGCACAGAGCAATTCGTTTAAC	42
0[132]	5[132]	GATAGGGGCCGGCGTTGCAACGAACTGAAAGGAGCCGGGAGA	42
0[174]	5[174]	CGAAATCAGCGGGCCGGCCTTAACCACCTTTGAGTAAGTTAC	42
0[216]	5[216]	TGGTTTGCGTAACCTAGTAATTAACACCCTCGTATCCTGAGC	42
0[258]	5[258]	TCACCGCCGCGTACGTCCATCATGAAAATTGAGGAAATTAAT	42
0[300]	5[300]	TTTTTCTCCTCGTTAAGTGTTAACCCTCCACTAACATGGAAA	42
0[384]	5[384]	GTCGGGAACGACGTGCGGGCCCTATTTTAAGGGTGGAGCTTC	42
0[426]	5[426]	CTAACTCGAAGCCATTCGCCAAGAGTCTTTTTAAACAGGATT	42
0[468]	5[468]	CGGAAGCCGCACGATTCCGGCAAACTAGTTATTTCTCATTTT	42
0[510]	5[510]	GTGAAATGACAATGGACGACAAAAGCCCCAAAAACGCTGTAG	42
0[552]	5[552]	ACCGAGCGCTCGCCGATGGGCTTGTAAATAAGCAAGTCTGGA	42
2[97]	11[104]	TTTTGACAAAGGGAACATTCAAAACGTCACCAATG	35
2[139]	11[146]	CCAGCCAAACGTGGAGAAAATTAGCGACAGAATCA	35
2[181]	11[188]	AAACTATGCTAGGGCGCAAAGCGTTTTCATCGGCA	35
2[223]	11[230]	CTTTGATACCACACCGTAGAATTGCCATCTTTTCA	35
2[265]	11[272]	AGAGTCTTATGGTTCTGGCATCACCGGAACCGCCT	35
2[307]	11[314]	TCCTGAGAGAATCAGGAAACCCACCCTCAGAGCCA	35
2[391]	11[398]	GATCGGTTGTAAAAGTTAGCGCAGACGATTGGCCT	35
2[433]	11[440]	AGCGCCAGGGTGGAACAAACTTAAAGCCAGAATGG	35
2[475]	11[482]	GCCAGCTCTTAAGTAATAGGAAGTAAGCGTCATAC	35
2[517]	11[524]	AGGGGACTCCCGCCACCCTCAGGTAATAAGTTTTA	35
2[559]	11[566]	TGGTGTACTGGAGTTTTAGTACCGTATAAACAGTT	35
5[91]	2[98]	GTCAGATGTTAATTAGTATAAAGCCAACTGAAGCCTTTGAATACCTACA	49
5[133]	2[140]	AACAATAGCAAGACGCCATATTTAACAAACGCGAGAAAACATATTACCG	49
5[175]	2[182]	AAAATCGTGGGTTATTTCGAGCCAGTAAGCAAGCAGATAAAAAGAACTC	49
5[217]	2[224]	AAAAGAATCATAGGTAAAGTAATTCTGTCCGTTTTACAGTGCAATACTT	49
5[259]	2[266]	TACATTTAGATTAAAGCTAATGCAGAACCGGGTATGCATCACGAGTAAA	49
5[301]	2[308]	CAGTACAATTAATTCTGAACAAGAAAAAAACCAATATCTGGTGCCAGAA	49
5[385]	2[392]	AAAGCGAAAATCAGCGGATATTCATTACAGGCGCATCTACAAGAAGGGC	49
5[427]	2[434]	AGAGAGTATGCTTTCAGTGAATAAGGCTTGTCGAAACAAGAGCAGGCAA	49
5[469]	2[476]	TGCGGATATACTGCAGTAAATTGGGCTTAACAAAGATCATATCACTCCA	49
5[511]	2[518]	CTCAACACAGAGGGTGTGAATTACCTTAAAAACACAGGAAGACAGTTTG	49
5[553]	2[560]	AGTTTCAAAAAACCCAGTCAGGACGTTGCGAAGGCATTTTGTGTCACGT	49
6[104]	9[97]	TATTTTAGAATATAATTATCAGATGATGCAATATTTTAAATCGATTTTT	49
6[146]	9[139]	TCCAATCACGGATTAAAGAAACCACCAGTAGCCCTGCGTTTTAGAGAGA	49
6[188]	9[181]	CTTAGGTCGCAGAGATTAATTTTAAAAGAGCAGAAAATCAGAAATTAAC	49
6[230]	9[223]	ATCAAAAGATGATGAAACAATTCGACAAGCCTGCATATTTTCGCTAATA	49
6[272]	9[265]	ATAGCTTAACAATTAATAGATAATACATATCTAAATAAACCAAATAATA	49
6[314]	9[307]	GCTATTATAAATCAAATATCTTTAGGAGAATCAATCAATAATACCGAAG	49
6[398]	9[391]	AAATCAAACCAGACAGGTAAAGATTCAATGAGAGAGACGGTCACAACTA	49
6[440]	9[433]	CCCTCAAACCTTTAAGAACCCTCATATAGGAGCAAATCCGCGATCTCCA	49
6[482]	9[475]	GCGTCCAGGCTTAGTGCGGGAGAAGCCTCATGTCATACAACGGGTTTAT	49
6[524]	9[517]	GTTTTGCTGTTTTATAAATCGGTTGTACCAAAAACTCATCTTGATACCG	49
6[566]	9[559]	CGACGATTTCCATAAGAATTAGCAAAATCGTTAATACCAACCCGCATAA	49
9[98]	0[91]	TGTTTAAAAGGGCGGCCCCCGAACAAGAGTCCACT	35
9[140]	0[133]	ATAACATAATCAATCGAGAAAAAAGAATAGCCCGA	35
9[182]	0[175]	TGAACACAAAGAAACGCTGGCTTTGATGGTGGTTC	35
9[224]	0[217]	TCAGAGATAGCAAACCGCCGCCAAGCGGTCCACGC	35
9[266]	0[259]	AGAGCAAAAAAGAAGCTTTGACAGCTGATTGCCCT	35
9[308]	0[301]	CCCTTTTACCAGAAGAGCGGGGGGCGCCAGGGTGG	35
9[392]	0[385]	AAGGAATCCTCATACGACGGCCTGCCCGCTTTCCA	35
9[434]	0[427]	AAAAAAACACCAGTTGTTCTTGCCTAATGAGTGAG	35
9[476]	0[469]	CAGCTTGCAAGCCCGTCCTTACACAACATACGAGC	35
9[518]	0[511]	ATAGTTGAACCGCCAAAATAATAGCTGTTTCCTGT	35
9[560]	0[553]	CCGATATCAGGAGGGACTCTAGAGGATCCCCGGGT	35
11[105]	6[105]	AAACCATAAGACAACGTCAAAGAGGTTTGCTCAACTCAAATA	42
11[147]	6[147]	AGTTTGCTTGTCACAAAAACATCTAAGACGCCAACATGCAAA	42
11[189]	6[189]	TTTTCGGACATATACCTGAACCCCAATATAAGAGACCTCCGG	42
11[231]	6[231]	TAATCAAAGTATGTGATAACCAAGCAAGCCAGACGTGAATTT	42
11[273]	6[273]	CCCTCAGAATACCCGAAACAACCAAGAAGCGCCTGCATAGCG	42
11[315]	6[315]	CCACCCTCAAAGTTTAAGAAAATGTAGATAATATCAATCGTC	42
11[399]	6[399]	TGATATTAGACAGCTGCGAATCGGAACGCCAAATCTGACCAT	42
11[441]	6[441]	AAAGCGCGTTTCGTGGCTCCATAAATTGTGCCCTGTGAATCC	42
11[483]	6[483]	ATGGCTTGGGATAGCTTTCGAAGCGCGAGAGATGGCTGGATA	42
11[525]	6[525]	ACGGGGTCCCTCAGCGCCGACATACACTTGCGATTAAAAGAA	42
11[567]	6[567]	AATGCCCCCGTACTATTCGGTGCCACTAGGAAGAATACCAGA	42
0[111]	5[111]	AGTTTGGATTTAGATGGAAATGGCTATTATTCCTGCAGTAAC	42
0[153]	5[153]	TAAATCAGGAAGGGGAACAATCGCCATTGCGGAACCGCCTGA	42
0[195]	5[195]	AATCCTGAAGTGTAGAGTAGACAGAGGTGAACGTTGCGAATT	42
0[237]	5[237]	GTTGCAGGCTTAATTTGTAGCCACGCTGACTTTACAAACAAA	42
0[279]	5[279]	CGGGCAACGAGCACGGCCACCCTTGCTGAGCCGTCTCATTTG	42
0[363]	5[363]	GCTGCATAACGCCACCAGCTGAATTAATCAGTCAATCAAATA	42
0[405]	5[405]	GCGCTCACAGTGCCCTGTTGGAGGCTATAATGTGTCGGAAGC	42
0[447]	5[447]	CTGGGGTCTAAGTGCGGAAACAATCGATAATTTTTATTGCTC	42
0[489]	5[489]	AATTCCAGTGCTGAAAGATCGGTACCCCATACTTTAGCTTAA	42
0[531]	5[531]	ATGGTCACCCCGCTCATCTGCTTGTATAATAAAGCAATATGC	42
0[573]	5[573]	AAAGACGTGATACCGGGATAGTAAAATTAAGGCAATAACAGT	42
2[118]	11[125]	ACGCTCAGCTTGACAGCGCCACGATAGCAGCACCG	35
2[160]	11[167]	ATATCCAAAGAAAGGTTTATTCTTTAGCGTCAGAC	35
2[202]	11[209]	CTTGCCTGCGGTCAGGTGGCATCATAGCCCCCTTA	35
2[244]	11[251]	TTAACCGGCGCCGCATTACGCAATCACCGGAACCA	35
2[286]	11[293]	TCAGTGAGTATAACATAACGGAGCCGCCACCCTCA	35
2[370]	11[377]	TATTACGGGGTTTTTTTGTCGTGACAGGAGGTTGA	35
2[412]	11[419]	TGCGCAAAAGCTTTATTCCACCACAAACAAATAAA	35
2[454]	11[461]	CTGGTGCGTTGTGAACACTGAAGTCTCTGAATTTA	35
2[496]	11[503]	CCTCAGGATTGTCAATTTTCATTGATGATACAGGA	35
2[538]	11[545]	AACCGTGTCTAATCACCGCCACAGTGCCTTGAGTA	35
2[580]	11[587]	CCGTAATGACAGTGTGTATCACCTGCCTATTTCGG	35
5[112]	2[119]	AGTACCTAACTTTTAGTAGGGCTTAATTCTCCCGAAATGCGCAGGAAAA	49
5[154]	2[161]	TTGCTTTAATGCTGATGTAATTTAGGCAGGCTTATCCGAACGGCTGGTA	49
5[196]	2[203]	ATTCATTTTTTTAAATATAAAGTACCGAGAATCATTCAGTATAACATCA	49
5[238]	2[245]	CATCAAGTCAATAGACGACAATAAACAACACTCATGCAGCAAACGCAAA	49
5[280]	2[287]	AATTACCTTGAAAATTTATCAACAATAGCTTTCCTAATATCATTTATAA	49
5[364]	2[371]	TCGCGTTTATTATAAAGAGTAATCTTGAAGGGAACAGGGTAGTCTTCGC	49
5[406]	2[413]	AAACTCCACGAGAAAACGTAACAAAGCTCCATGTTTTGCCTGTTCAGGC	49
5[448]	2[455]	CTTTTGATATTCATACGAGAAACACCAGGTATCATAATCGTAACCGCTT	49
5[490]	2[497]	TTGCTGAGTTTAGATTTAATTTCAACTTCAGCGATAATCAGAGTATCGG	49
5[532]	2[539]	AACTAAACTTTTGCTTAAGAACTGGCTCAAAGAGGATTTAAAGCATCGT	49
5[574]	2[581]	TGATTCCCCTCGTTAAATCTACGTTAATGTAAAATAATTTTTGGATTGA	49
6[125]	9[118]	GCGAGAATTTACATGGAATTATCATCATAGTCTTTCTTGCGGAATGAAA	49
6[167]	9[160]	ATATGTAGAATACCAACATTATCATTTTAAAAATACCGGTATGGGAAGC	49
6[209]	9[202]	GACTACCTCAATTATAAATCCTTTGCCCGAGGCGGTACCGCGAAAGTCA	49
6[251]	9[244]	AGAAGAGAAAACAATTTAGAAGTATTAGAGAGCCACGAGAACCACAAGA	49
6[293]	9[286]	AGAATCCTTTTTTAAACTAATAGATTAGAACCTCATATCATTTGAAATA	49
6[377]	9[370]	CCCTGACTTAATTCAGAAAGGCCGGAGAGCCGGAGCGAACTGGCGGAGT	49
6[419]	9[412]	TCAGAAAAACAGGTTGCAATGCCTGAGTCAGGTCAACTTAGCAATAATT	49
6[461]	9[454]	TCATAAATAAGAGGAACGCAAGGATAAAGAACGGTCGCCTGAAAAGGAG	49
6[503]	9[496]	GTAAAATATATAATATTATGACCCTGTAGGTTGATTATACCAGGTGAAT	49
6[545]	9[538]	CGAGAGGGTACGGTTAAAGCCTCAGAGCAGCAAATCAAAAGAAATGACA	49
6[587]	9[580]	TCATAACCAATTCTTAAATCATACAGGCCGCATTAACGTAATCGCTGAG	49
9[119]	0[112]	ATAGCAGGTTTACCGGGGAAATTGAGTGTTGTTCC	35
9[161]	0[154]	GCATTAGGGAATAACGAAAGGGGCAAAATCCCTTA	35
9[203]	0[196]	GAGGGTAACATAAACGCTGCGCCCCAGCAGGCGAA	35
9[245]	0[238]	ATTGAGTACTCCTTTACAGGGCTGGCCCTGAGAGA	35
9[287]	0[280]	GCAATAGCGCAATAGTGCTTTTTTCACCAGTGAGA	35
9[371]	0[364]	GAGAATACTAAAGTCCCAGTCAACCTGTCGTGCCA	35
9[413]	0[406]	TTTTCACCTGTAGCCTCAGGAACATTAATTGCGTT	35
9[455]	0[448]	CCTTTAATACCGTAATTCATGATAAAGTGTAAAGC	35
9[497]	0[490]	TTCTTAACACCCTCACCTTATTGTTATCCGCTCAC	35
9[539]	0[532]	ACAACCACCTCAGATATTTACTCGAATTCGTAATC	35
9[581]	0[574]	GCTTGCAGAATAGGCGGCCCTCTTGTTACCTCGAT	35
11[126]	6[126]	TAATCAGTCATATGCCTTTACAGCGAACGAGAATCAAAGAAC	42
11[168]	6[168]	TGTAGCGACACCACACGGGAGTATAGAAGAGGCATTATAACT	42
11[210]	6[210]	TTAGCGTAATACATATTGAGCATCGTAGCAAAAGGTCTGAGA	42
11[252]	6[252]	GAGCCACGATTAAGTAAGCCCAGTACCGCATGTTCGACGCTG	42
11[294]	6[294]	GAACCGCGAGGAAACTATCTTCGGCTGTATAAGTCTTCCCTT	42
11[378]	6[378]	GGCAGGTTAACGATGAAAGGAAATCATACAAGAACGTCTTTA	42
11[420]	6[420]	TCCTCATACAACGCGTTGAAAACCTGCTGCTCATTAAACAGT	42
11[462]	6[462]	CCGTTCCACCCATGTTGTATCGAGATTTAACGAGTGGAATCG	42
11[504]	6[504]	GTGTACTGAGCCACACAGCTTTGACCCCTAATCATGGTAATA	42
11[546]	6[546]	ACAGTGCCCGCCACTCGCCCATAAAACGATTATACAAAATAG	42
11[588]	6[588]	AACCTATTAGCCCGGGGAGTTTAAACGGAAAACGAAACACTA	42

biotin-modified
GTCAAAGGGCGAAAAACCGTCTCCCTCC-Biotin
ATTGTCAACCTTATGACAATGTCCCGCCATCTC-Biotin
Biotin-ATTCTCATACCTAATATTATAGTCAGAAGCAAAGC
Biotin-ATATCTAATCCTCTGAGATTTGTATCATCGCCTGA

digoxigenin-modified
Dig-TATTACCCACCCTACCCTAAAACATCGCCATTAAAAA
Dig-TACCACAATTCATTTTTGAATTACCTTTTTTAATGGA
CCAGCGCCAAAGACAAAAGGGCGACATTCATCTTCTA-Dig
AGGGTTGATATAAGTATAGCCCGGAATAGGACTCCTT-Dig
